# Information of Complex Systems and Applications in Agent Based Modeling

**DOI:** 10.1038/s41598-018-24570-1

**Published:** 2018-04-18

**Authors:** Lei Bao, Joseph C. Fritchman

**Affiliations:** Department of Physics, The Ohio State University, Columbus, Ohio, USA

## Abstract

Information about a system’s internal interactions is important to modeling the system’s dynamics. This study examines the finer categories of the information definition and explores the features of a type of local information that describes the internal interactions of a system. Based on the results, a dual-space agent and information modeling framework (AIM) is developed by explicitly distinguishing an information space from the material space. The two spaces can evolve both independently and interactively. The dual-space framework can provide new analytic methods for agent based models (ABMs). Three examples are presented including money distribution, individual’s economic evolution, and artificial stock market. The results are analyzed in the dual-space, which more clearly shows the interactions and evolutions within and between the information and material spaces. The outcomes demonstrate the wide-ranging applicability of using the dual-space AIMs to model and analyze a broad range of interactive and intelligent systems.

## Introduction

Information is a broadly used term representing the knowledge and beliefs about aspects of the physical world. When describing a system’s states and dynamics, the current definition of information is often based on a probabilistic configuration of the system, which doesn’t describe actual local interactions. In addition, a system with intelligent agents can carry information that significantly influences the behavior of the system, while the information itself can evolve within its own space independent of the physical entities. Therefore, it is beneficial to examine the existing framework of information to more clearly represent internal interactions and to apply such information in modeling complex systems.

This study is motivated by a physics example of a 2D ideal gas consisting of $N$ particles in a square container. The particles can elastically collide with themselves and the walls of the container. Assume that all particles are initially at rest and are set in motion by giving energy to a single particle, causing it to have initial momentum ${\overrightarrow{p}}_{0}$ and kinetic energy ${p}_{0}^{2}/2m$. This energy and momentum is then dispersed throughout the system by many elastic collisions. Throughout this process, the total kinetic energy ${E}_{T}={p}_{0}^{2}/2m$ and the system’s momentum (vector) ${\overrightarrow{p}}_{sys}={\overrightarrow{p}}_{0}$ are always conserved.

However, consider a non-traditional measure, the total absolute momentum, ${p}_{T}={\sum }^{}|{\overrightarrow{p}}_{i}|$, which is the sum of the magnitude of each particle’s momentum. It is found that ${p}_{T}$ would increase from the initial value of ${p}_{0}$ to an equilibrium state maximum (SI-S1.1). For the ideal gas, which follows the Maxwell-Boltzmann distribution at equilibrium^1^, ${p}_{T}$ is proportional to the square root of the total number of particles in the system (SI-S1.2):

1 $${p}_{T}=\sqrt{\frac{\pi }{4}}{p}_{0}\sqrt{N}.$$

Results of an ideal gas simulation match the theoretical relation. Similar relations hold for a 3D ideal gas (SI-S3) as well as a gas with a heterogeneous mixture of particle masses (SI-S1.3).

What is interesting is that with conserved total momentum (vector) and kinetic energy, the total absolute momentum at equilibrium will increase with the number of particles in a system. Therefore, certain information is embedded in the total absolute momentum that cannot be completely described by momentum and energy conservations. Since ${p}_{T}$ is a result of interactions between particles, it must represent features of such interactions: from the initial to the equilibrium state, the particles in the system increase their overall mobility causing the system to be more interactive and disordered and ${p}_{T}$ provides a measure of such mobility.

Similarly, one can inspect another variable, the rate of collisions between particles, which provides a measure of the interactivity of a system. The particle-particle collision rate ${f}_{p}$ is found to be proportional to ${N}^{3/2}$, again an increasing trend of change with the number of particles in the system (Eq. 2, SI-S2.2).

2 $${f}_{p}=\frac{\,2R\,\sqrt{\pi }}{{a}^{2}\sqrt{2}}\frac{{p}_{0}}{m}\,{N}^{\frac{3}{2}}=\frac{\,2R\sqrt{2}\,}{{a}^{2}}\,N\frac{{p}_{T}}{m}.$$

Prior to equilibrium, total absolute momentum and the collision rate evolve in a predictable sigmoidal fashion with time (SI-S1.5, S2.2).

Regarding the disorder of the system, it is helpful to examine entropy, which is defined to describe the scale of disorder of a system. The entropy of a system has been defined both classically and statistically (SI-S2.1). The general statistical definition by Gibbs gives^2^:

3 $$S=-\,N\,{k}_{b}{\sum }^{}{P}_{i}\,\mathrm{ln}({P}_{i})$$

with ${P}_{i}$ being the probability of a particle existing in the ${i}^{th}$ energy state. For the ideal gas example, the system’s initial entropy with only one particle moving at ${\overrightarrow{p}}_{0}$ is ${S}_{0}\approx {k}_{b}lnN$. The system’s entropy then increases through collisions and reaches an equilibrium maximum. The equilibrium entropy varies with ${p}_{0}$ and $N$, and increases nearly linearly with $N$ (SI-S2.1).

It is interesting to see that for a system having identical initial momentum (vector) and energy, $S$,$\,{p}_{T}$, and ${f}_{p}$ at equilibrium all increase with the number of particles in the system. Among the three variables, ${p}_{T}$ and ${f}_{p}$ are directly connected to local interactions. That is, ${p}_{T}$ and ${f}_{p}$ can be obtained with a bottom-up approach by accumulating the individual particles’ momenta and collision rates to produce ${p}_{T}$ and ${f}_{p}$ of an interested domain, which doesn’t need to be pre-defined. As a result, the total absolute momentum and collision rate are measures directly reflecting information of local interactions.

In contrast, the entropy gives a measure of the degree of disorder of a system based on a pre-defined probabilistic framework^3^. That is, to determine the probability distribution of the states of a system, the entire system and its possible states need to be known. This reflects a top-down process that begins with defining the entire macroscopic system and down to the probabilities of specific states determined based on the defined system. For the entropy measure, a bottom-up approach is impossible. The information of individual particles of a system cannot be used to calculate the entropy without knowing the complete probabilistic configuration of the entire system. Therefore, the entropy is a macroscopic measure of the system that doesn’t directly reflect local interactions.

Generalizing from the specific measures discussed above, it is useful to distinguish a category of information that measures features of local interactions within a system, which can be defined as “local information”. The actual formalism of local information is dependent on the actual system and its interactions, and therefore, should be determined in each case.

To study the proposed local information, it is helpful to review the existing information theory to examine the features of its representation. A large component of the current definition of information uses stable states of a system as symbols to encode information that is used for external purposes such as in applications of digital media storage and communication^4–6^. The information is defined by external agents and used for representing external processes and operations. Internal states of the system are used to encode the information but are not represented by such information. The system’s states act merely as the medium to store and transmit information; therefore, this type of information is fundamentally external to the system.

In general, any system that has distinct stable states can be used to encode and store information. The states of a system exist in a probability distribution, such as the Maxwellian speed distribution of particles in an ideal gas. To define this distribution, all possible configuration states of a system need to be known, which form a normalized complete set that can be used to define the probability of the occurrence of a specific state.

Following the Shannon and Gibbs information entropy definitions, the scale of information that can be encoded in a state is the log of the inverse of the probability of the state’s occurring, ${h}_{i}=-\,\mathrm{log}({p}_{i})$. As a result, the equilibrium states, which are very likely to occur without any artificial priming (more disordered), would encode minimal information. In contrast, low probability states, which are much less likely to occur without significant priming (less disordered), can encode larger amount of information. Once established, a low probability state can be easily detected, with which information can be robustly encoded and retrieved.

Operationally, a system is defined by an observer and may have an arbitrary content domain. Depending on the goal of the observer, a system can contain many different constituents from particles in a gas to galaxies in the universe, and systems themselves can contain sub systems. A system and its probabilistic states are both defined by an observer and are therefore fundamentally external to the system’s constituents.

Apparently, the current information theory uses a system’s states as symbols for encoding information. The encoding properties of a system such as the capacity and uncertainty are based on the system’s distribution of probability states, which are defined by the observer and are external to the system. The information encoded doesn’t describe the internal properties of the system’s constituents. Therefore, the information defined in the current information theory is considered as a category of “external information”, which is defined with a system’s states but used for representing entities external to the system.

Similarly, entropy also follows the same kind of probabilistic definition, which relies on an externally defined probability framework. As discussed previously, entropy does not directly describe local interactions but provides a measure of the disorder of the system based on the externally defined probabilities of the system’s states, which are the outcomes of the local interactions. This is different from the external information discussed above. Meanwhile, entropy does not provide direct measures of local interactions, and therefore, is not a measure of local information. It uses an externally defined probability framework to describe the disorder of the system at the system-wide level. As a result, entropy can be considered as a measure of “system-state information”, representing statistical features of a system. In addition to entropy, the speed distribution of an ideal gas is another example of “system-state” measures.

Summarizing the discussion, three categories of information can be broadly defined: (1) local information that describes a system’s internal local level properties, (2) external information that is encoded based on a system’s states but is used for representing entities external to the system, and (3) system-state information that is defined based on an externally established probability framework to provide statistical descriptions of a system.

Distinguishing the different types of information will clarify the existing conceptual understanding of information. For example, both entropy and the current definition of information do not represent properties of a system’s internal constituents and their interactions. Furthermore, existing interpretations of entropy and information do not explicitly distinguish between external and internal properties of a system, which can cause ambiguity in the conceptual basis of these measures and limit their uses in modeling the system’s behavior.

The local information is more directly related to internal interactions and will be examined closely in this study. The concept will also be applied to develop information based agent models to study large-N interactive systems. In the ideal gas example, local information about the particle-particle interactions can include individual particles’ momenta and frequencies of interactions, which are measured with ${p}_{T}$ and ${f}_{p}$. Since there exist many types of interactions, the actual measures of local information are system and interaction dependent, and need to be defined and formulated for each specific case. However, this doesn’t limit the exploration of some general features of local information.

In the ideal gas example, the interactive forces between two particles involved in an elastic collision are symmetric (equal and opposite) and the changes of the momentum for the two particles are also equal and opposite ${\rm{\Delta }}{\overrightarrow{p}}_{i}={\int }^{}{\overrightarrow{F}}_{ji}dt=-\,{\rm{\Delta }}{\overrightarrow{p}}_{j}=-\,{\int }^{}{\overrightarrow{F}}_{ij}dt$. As a result, the total vector momentum is conserved (${\rm{\Delta }}{\overrightarrow{p}}_{i}+{\rm{\Delta }}{\overrightarrow{p}}_{j}=0$). When only keeping the magnitude (removing spatial vector relation), the absolute momentum is in general not conserved (${\rm{\Delta }}{p}_{i}+{\rm{\Delta }}{p}_{j}\ne 0$). It appears that the conservation laws (vector momentum and energy) apply to materially observable measures measured with spatial properties (${\rm{\Delta }}KE={\rm{\Delta }}K{E}_{i}+{\rm{\Delta }}K{E}_{j}={\int }^{}{\overrightarrow{F}}_{ji}\cdot d\overrightarrow{x}+{\int }^{}{\overrightarrow{F}}_{ij}\cdot d\overrightarrow{x}=0$). Meanwhile, information measures such as ${p}_{T}$ and ${f}_{p}$, which are measured with only time property or with spatial property removed, would follow different relations.

It is then helpful to distinguish between information and material measures by defining two separate spaces: the information space and the material space. This will be referred to as a dual-space framework that can be used to model and analyze complex systems. In the ideal gas example, vector momentum and kinetic energy are material space measures that follow the conservation laws, while ${p}_{T}$ and ${f}_{p}$ are information space measures that don’t follow conservation laws. Broadening into socio-economic systems, the information space measures of agents of a system can include education, knowledge, news, etc., which are unbounded and constantly evolving. On the other hand, material space measures can include money, land, water, etc., which are often conserved in a short period of time.

For socio-economic systems, agents of a system can also have intelligent features as a part of their local information such as the agents making decisions on who to interact with and how to interact. For intelligent agents, their local information can evolve and accumulate by itself without a strict dependence on material based interactions. Systems with intelligent agents can be defined as intelligent systems. An intelligent system doesn’t necessarily have an equilibrium state in information space, which can constantly evolve even when materials are conserved. On the other hand, physics systems can be considered non-intelligent, with the agents of the system not making individual decisions during interactions but simply following physics rules. In such systems, local information is directly related to material space measures and reaches maximum at equilibrium.

Synthesizing the discussions, a number of features of local information can be postulated:

1. Local information can exist and change without strict dependence on material based conservation laws.
2. For a non-intelligent system, before reaching equilibrium, the total local information and its change are non-negative.
3. An intelligent system doesn’t have a complete equilibrium state. Local information always increases even when materials are conserved.
4. Local information of a system increases when materials and interactive agents are added to the system (e.g., adding energy to a system or mixing two systems).
5. Information measures can interact with material measures.

### Dual-space Modeling of Intelligent Systems

Agent-based models (ABMs) have been widely used in modeling socio-economic systems, such as the economy and stock market^7–13^. Among the existing ABMs, information has long been used as conditions of the agents’ decision-making rules. Typically, decision-making is structured with conditions and actions such as the simple if-then process (if condition-x then action-y). A concrete example could be that if the price is below some amount (an information based condition), then buy (a material based action). Here the conditions are the outcomes of information processing, while the actions are the material based events. For information to function in an agent’s decision-making, two layers of information processing can be considered. One is the abstract layer of cognitive or intelligent processing that can be domain general and accounts for the intelligent features of the agent. The other is the concrete layer of actual conditions, which are the outcomes of the abstract intelligent processing expressed within the specific contexts of a model. In most previous research, the information is primarily used in the form of concrete conditions of decision-making rules with varied complexity. Rarely is the evolution of information itself studied; instead, focus is placed on the decisions made and consequences of these decisions. As a result, the abstract layer information processing and evolution that lead to the concrete conditions are often not included as part of the model.

In this study, the emphasis is on the abstract layer information evolution such as intelligent learning and cognitive development, which can be unrestricted with specific material based context. Building on the concept of separating the information and material spaces, a dual-space agent modeling framework is developed by explicitly defining an information space to include the entire spectrum of concrete and abstract information elements and processes. The framework is referred to as agent and information modeling (AIM) to emphasize its focus on the independent dynamics of information. Following the AIM framework, targeted models can be developed to study different socio-economic systems. In general, the information space would contain measures of all categories of information including local, external, and system-state information that may exist in a complex system and influence the system’s behavior.

The general formulation of AIM builds on variables and rules in dual-space and allows interactions within and between the two spaces instead of in a single material space. Information interacts with the system in multiple ways. In general, information influences the material space interactions, which in turn causes agents involved to gain new information. Intelligent systems allow information to evolve within its own space without strict dependence on material space activities and rules. It is assumed that information will never decrease; agents may fail to access a piece of acquired or developed information but will not lose it. However, certain information may inhibit or bypass the influence of other information.

AIM interactions can be expressed as a recursive system of equations (Eq. 4). For a system at time $t$, the material space is represented by $M(t)$ and the information space is represented by $I(t)$:

4 $$\begin{array}{c}M(t+{\rm{\Delta }}t)=F(M(t),I(t))\,\\ I(t+{\rm{\Delta }}t)=f(M(t),\,I(t)).\end{array}$$

Here $F$ and $f$ are system dependent functions, which define (1) how information influence material interactions by deciding which agents can interact and how the interactions are carried out; (2) how agents change their information during interactions; and (3) how information itself may change with and without material space interactions (SI-S4.1).

For a non-intelligent system such as the ideal gas, the information measures depend only on the material space; i.e. information is either directly related to the material space or is unable to evolve independent of the material space. In contrast, an intelligent system consists of agents who can learn from interactions and can have unbounded information. This culminates in a system of equations containing functions within functions, which lead to non-linearity and emergent behavior in both the material and information spaces.

This paper seeks to emphasize the theoretical formalization of including information as a separate space in intelligent systems. Economic models are used as baseline examples because of the wealth of literature available with established results over the last several decades. The focus of models in this paper is then on how to use information, how the inclusion of information affects a model, and how to utilize the information space for analysis and presentation. To demonstrate the benefits of AIM, it is useful to discuss the types of information usage in previous ABMs, and in what ways AIM will compare or diverge from them.

Non-ABM economic models have studied information beginning around the early 1970 s with the information revolution and many examples have been demonstrated in textbooks and literature such as the effects of asymmetric information^14–19^. A prime example is the model proposed in Akerlof’s “The Market for Lemons”^18^ which uses information about the quality of a traded product by directly coding this into the perceived or claimed value of the product. As such, information directly translates into the condition of a price decision-making rule in an if-then format (if an agent knows the quality of the product, then it trades with actual price). All interactions are conducted within this ruleset with minimal assumptions about the intelligence of the traders^20^ and there is no learning or gaining information through these interactions. Therefore, this is fundamentally a model of non-intelligent system, different from the AIM models.

There are also many ABM studies, which use information in ways similar to the non-ABM economic models^8,21–28^. For example, Glosten and Milgrom’s^22^ or Jacklin *et al*.’s^23^ stock market models use information such that informed agents know the expected price of a stock after a time period and un-informed agents do not. This creates an asymmetry in information and allows the informed agents to utilize an insider trading strategy to maximize profits. The trading mechanisms of these ABMs including the information definition, knowing or not knowing the value, as well as using information in determining trading rules, are fundamentally identical to the non-ABM models discussed above. There are also variations of information usage in ABMs including Conway’s Game of Life^24^ and the Sugarscape model^25^, which use the information as a descriptor of the material space. Some other ABMs allow more flexibility in using information measures, such as a choice from among a set of strategies, but these options are often bounded by pre-defined possibilities^26–28^. Here, the information is again used as conditions in decision-making rules or as selection criteria for choosing certain subset from a collection of pre-defined rules. Within these models, information itself is not designed to allow independent evolution. Therefore, these systems are generally non-intelligent and their information use differs from the AIM models.

In the literature, there are very limited studies that use information in more intelligent ways such that information is allowed to evolve separately from the material space. A rare example is the work by Pastore, Ponta, and Cincotti^29–31^. They studied markets using information as sentiments about the market, which determine how much of an asset an agent would buy or sell. While sentiments reflected perceived valuation of the asset, it was free to evolve based on an individual agent’s view of the market and influences of other connected agents. The implementation of the sentiment shows a step towards the dual-space AIM framework such that the information variable can evolve independent of the material space.

In the literature, there is an obvious trend towards more intelligent and sophisticated ways of applying information to study complex systems. As suggested by Gallegati^32^, moving forward in ABM, “requires ‘new’ physics, which is based on non-ergodicity, on social dynamics whose elementary constituents are heterogeneous interacting social agents linked by networks”. This suggestion is operationally accomplished in some of the existing models at various levels. However, substantial advancement often requires a conceptual change at the theoretical level, which is the main emphasis of the dual-space AIM framework. By adding the information space to form the dual-space structure, AIM models require the explicit definition of an independent information space and its evolution mechanism. The information space becomes a necessary modeling and analytic dimension rather than a collection of conditions of material trading rules. A form of rules is still required to model a system, but within AIM they will now evolve with the evolution of the information space. As an independent dimension, information is also used in AIM for data analysis and presentation, which were rarely utilized in previous models.

To explore the features of AIM and compare them with previous models, three economic examples are examined: (1) information space interactions are added to a traditional money distribution ABM to explore the basic dynamics of a simple AIM model; (2) evolution trajectories of selected agents are examined using AIM to study and predict behaviors of individual agents under various conditions; (3) AIM is applied to study an artificial stock market to explore the emergence of economic collapse and recovery.

### Model 1: The Crescent of Money Distribution

Among others, Dragulescu and Yakovenko^33^ and Chatterjee and Chakrabarti^34^ studied ABMs for money distribution. Their systems didn’t include any explicitly defined information variables and agents exchanged money according to pre-defined trading rules (a random selection in this case). The simulations ran with the goal of finding an equilibrium state of the system, which produced the distributions of the variables studied. Effects of asymmetric information have also been explored in money distribution models^35^. Typically money distribution models approach this choosing a portion of money to save^36^ or similarly how much to risk^37^. Kinsella, Greiff, and Nell^38^ examined local interactions of heterogeneous agents and asymmetric information and found power-law relations to emerge. However, the use of information is primarily as conditions in trading rules; essentially each new system requires creating a new set of rules. None of these models allow evolution of the information itself. Therefore, these ABMs are non-intelligent systems with rules and operations exist as part of the material space.

On the other hand, the dual-space AIM models implement an information space that can evolve independent of the material space. Every interaction conserves material space just as the money conservation in the existing ABMs. However, the information space grows continuously as a result of agents gaining information through trading and the gained information influences future interactions. In an AIM model, rules defining the interactions can stay simple and fixed while the configuration of information variables can change dramatically to allow modeling vastly different behaviors. This provides a broader applicability for an AIM model and reduces the need to make a new model for each of slightly varied systems as is necessary with some of the ABMs.

The example below shows an AIM model for money distribution (SI-S4.2). In this model, the information space includes a general measure of knowledge and education that contribute to the intelligent capability of an agent for doing profitable trade. The material space trading rule is identical to the ABMs. The winner of a trade gains the money that the losing agent loses. The differences are that the AIM model picks the winners of trades through a biased random process with a strong preference for higher information agents. However, if the information difference is too large, low information agents may reject the trade, knowing that they are likely to lose. During a successful trade, both agents gain information with the winner gaining a larger amount than the loser. For rejected trades, the money doesn’t redistribute but both agents gain information with the agent rejecting the trade gaining a larger amount.

**Figure 1:**
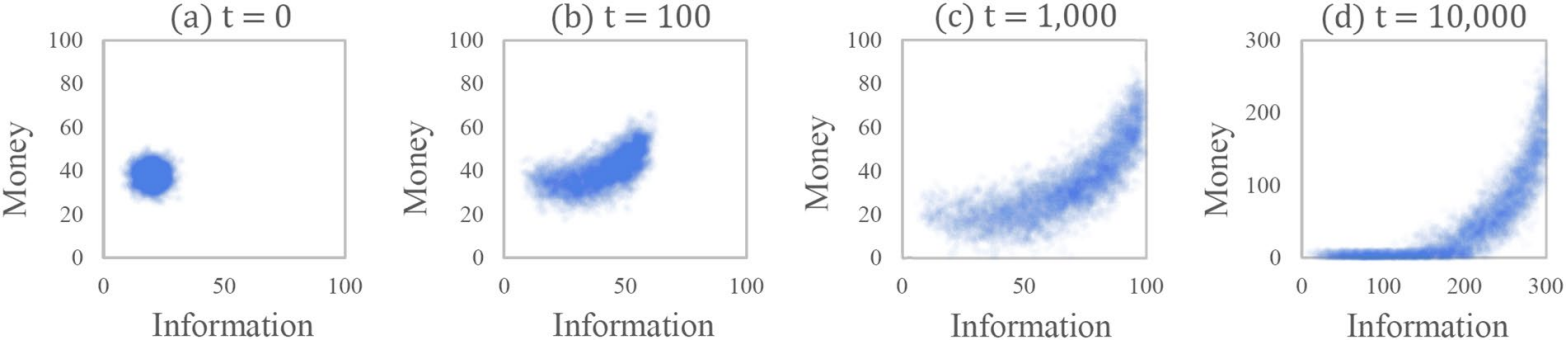
The crescent shape forms quickly from an initial normal distribution. (**a**) The initial distribution (Gini index $G=0.06$). (**b**) 100 time-steps: beginning of the crescent ($G=0.11$). (**c**) 1,000 time-steps: fully developed crescent ($G=0.26$). (**d**) 10,000 time-steps: boomerang shape emerges after many time-steps ($G=0.61$). All diagrams are scaled such that 100 money or information is the maximum value of money or information for a single agent in the crescent distribution shown in part (c). Note that the axes on the boomerang distribution extend beyond 100.

In AIM models, the information space is also used as a dimension in data analysis and presentation. With the addition of the information space, the money distribution results are shown in a 2D money-information diagram (Fig. 1). Regardless of the initial conditions, the distribution quickly transitions into a crescent moon shape as shown in Fig. 1a–c. After a long time, the crescent thins out into a boomerang shape with most of the population having low information and low money and a small number of agents having high information and high money, representing an economy with high inequality (Fig. 1d, Gini index $G=0.61$). This pattern generally matches those seen in real economies when comparing education level and income: higher levels of education typically result in higher incomes^39^.

**Figure 2:**
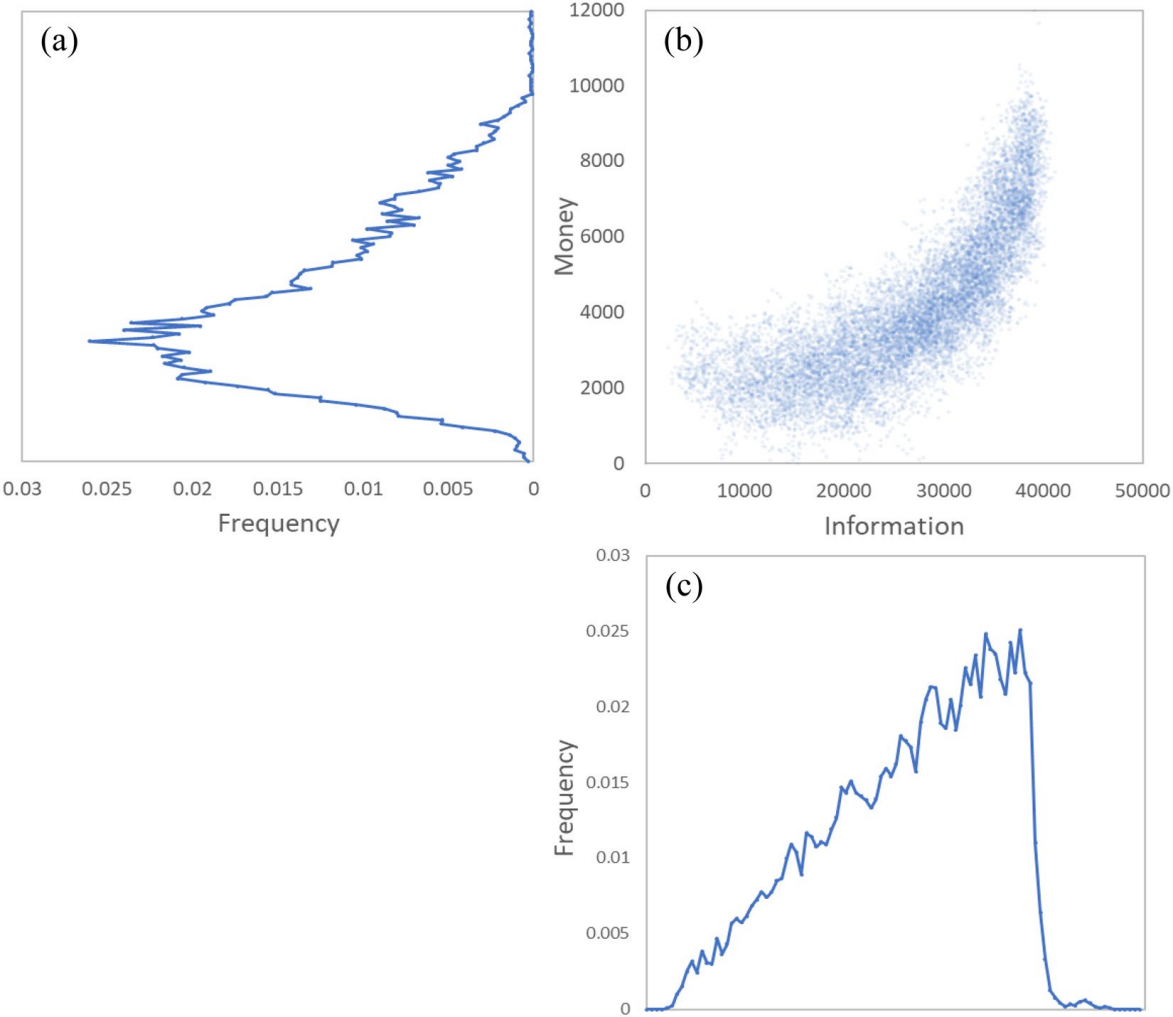
The crescent shaped information-money distribution with its separate information and money distributions. Information and money are plotted as percent of the maximum single agent’s information and money at $t=1,000$. This figure demonstrates one method of analyzing and representing the dual-space.

**Figure 3:**
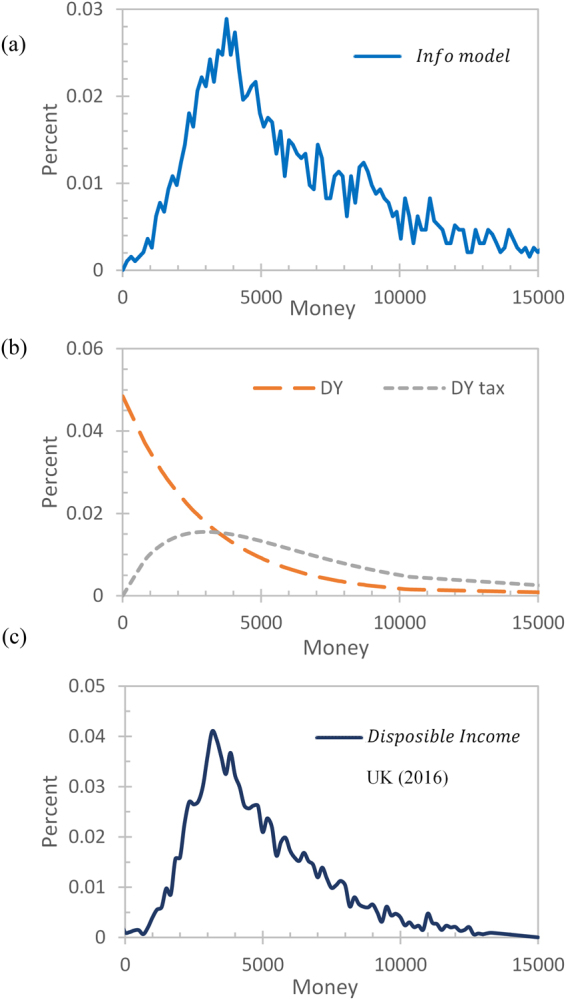
Money distributions. (**a**) AIM, (**b**) the Dragulescu & Yakovenko^33^ models (DY: the basic ABM model; DY tax: basic ABM model with tax rules), (**c**) the disposable income distribution from the UK 2016^41^. The percent of the population with money m is plotted. The DY model and UK data are scaled to have an average amount of money equal to that of the AIM model (5,000).

The crescent moon shape distribution is an interesting intermediate stage of the evolution that can be considered as a stage of balanced middle-class population with a Gini index of 0.26, favorable for a healthy low inequality economy. A 2D crescent along with its money and information distributions are plotted in Fig. 2 for a closer inspection. The information distribution has a long nearly linearly increasing left tail before peaking and sharply falling off after. The money distribution has a shifted gamma-like distribution with a long right tail that can be fit by a power law distribution. This matches well with the real-world distributions, which typically have a small portion with near zero money, a major peak before the median, and the existence of a power law tail for high money values^40^. Specifically, this model is strikingly similar to the UK disposable income distribution from 2016 (Fig. 3a,c)^41^.

Existing ABMs also produced distributions comparable to real-world economies at varying levels. Basic ABMs often failed to produce close matches to the real-world results with a peak at zero money and exponential decays at larger money values (Fig. 3b, DY). When adding rules such as taxation or savings, the distributions became gamma-like with a non-zero peak and a power law, resembling the real-world distributions (Fig. 3b, DY tax)^33^.

The main benefit of the dual-space AIM model is that the outcomes are emergent as a result of interactions between the information and material spaces without the need of additional hard-coded rules such as taxation and savings. The simulation results of the AIM model demonstrate that the addition of the information space allows pre-established rules to evolve over time, instead of being a static function of the state of the material space. The intelligent system operates as a function of functions, allowing greater non-linearity and opportunity for emergent behavior. In addition to modeling, the results are also analyzed with two spaces allowing clearer presentation and identification of possible patterns, relations, and attractors. Based on this study, it can be implied that the dual-space structure with an independent information space may be a key improvement in modeling and analyzing intelligent complex systems.

### Model 2: Lottery, Grants, and Creativity

Evolutions of individual agents may be of primary interest when searching for optimal conditions for these agents to succeed. For example, policy makers will want to understand the possible effects of certain social-economic stimuli that can be implemented with disadvantaged population groups to improve their economic standing. Meanwhile, venture capitalists will be interested in evaluating what conditions would give an agent optimal chances for success in order to obtain best investment returns.

Existing ABMs do not typically study the evolution of individual agents but focus on the group behavior of a system at equilibrium. In addition, for a simple rule-based system, non-linear coupling may not occur and an individual agent’s behavior is often predictable and less interesting. In contrast, the AIM dual-space method allows complex non-linear coupling between the information space and material space, which makes an agent’s behavior unpredictable with many local attractors of interactions between the information and material measures. The examples explored in this study show how in a simple form one can configure a money distribution model with reconditioned money or information variables to examine the evolution of an individual agent. Many different conditions can be easily explored to produce statistics of interesting evolution pathways and states, which can be further analyzed to determine the optimal conditions for an individual’s success and to predict the likelihood and the scale of such success.

In a real economic system, targeted stimuli can be implemented to substantially change an individual agent’s economic status while minimally affecting the system. Examples of these stimuli can come in forms of grants (large money given to a high information agent such as a start-up entrepreneur), lottery (large money given to a lucky random agent), and creativity (large information boost to a random agent such as someone creating a marketable new idea).

The AIM simulation was implemented as an extension of the dual-space money distribution model discussed above. The crescent-shaped distribution from the first model was used as the system’s background, which was fixed. Each stimulus was applied to one representative agent. Three types of agents were conditioned, lottery (L), grant (G), and creativity (C). A simulation run included only one of such agents at a time and there were thousands of runs of the same agent with identical initial conditions to produce statistical results of an agent’s evolutionary pathways. In a simulation run, the system’s evolution was “frozen” so that the non-conditioned agents wouldn’t interact among themselves. The only agents to interact were the representative agent and agents it directly interacted with. This was done to control variables in order to avoid confounding effects from the system’s own evolution.

**Figure 4:**
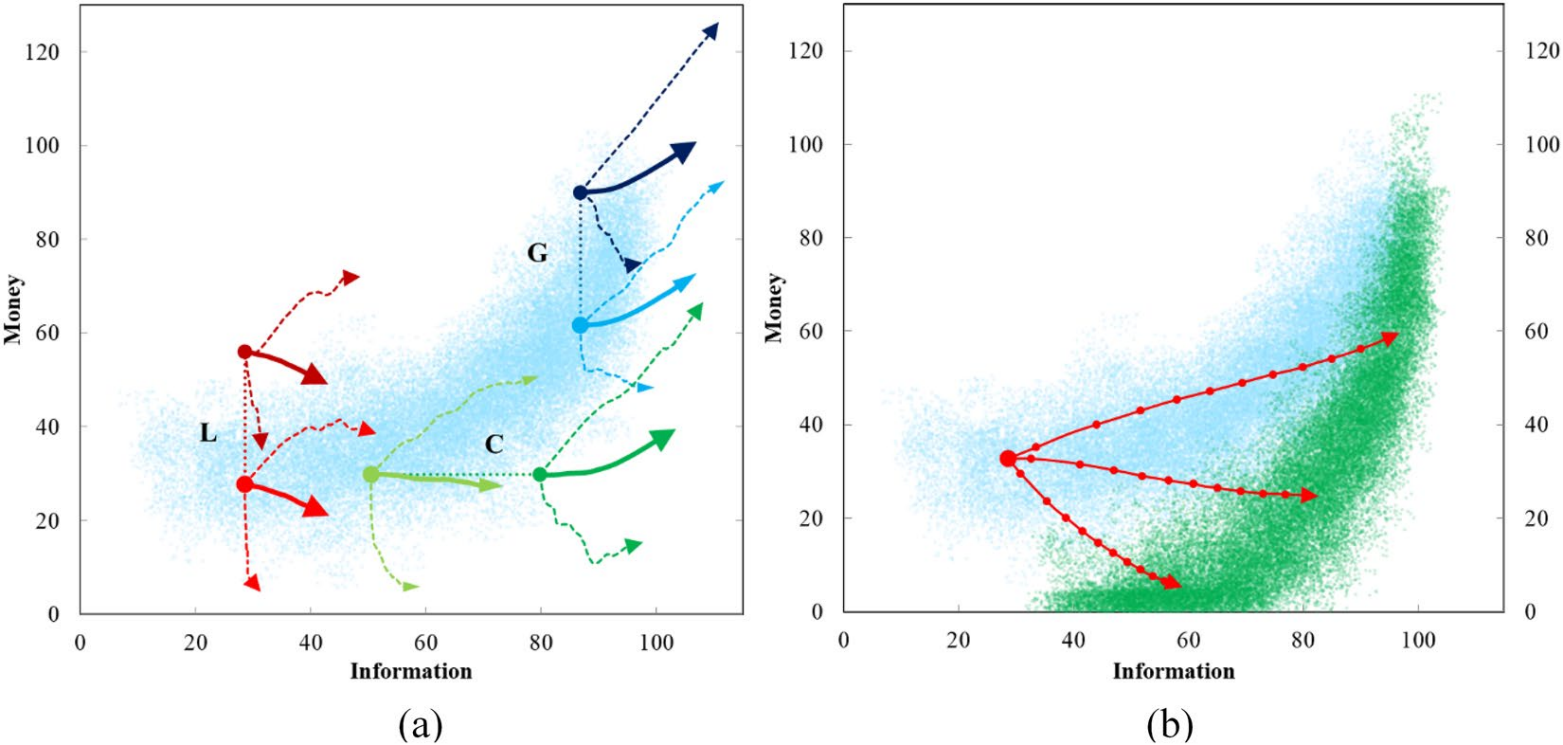
Agent trajectories. (**a**) Trajectories of agents under grant (G, blue), lottery (L, red), and creativity (C, green) before and after stimulus are applied. Average, minimum, and maximum trajectories are shown for each agent. (**b**) Possible long-term behavior of a single agent is plotted. The three red lines represent average trajectories of the agent when evolving into low, mid and high classes. The scale in these diagrams is percent of the original maximum information (horizontal) and money (vertical) of an individual agent in the crescent.

The evolutions of the three types of agents were recorded for their first 1,000 trades and the results are plotted in Fig. 4a. The evolution pathways of both the original agents (without stimulus) and the conditioned agents are plotted (six agents in total). Each agent’s initial condition and the effect of stimulus in the crescent are marked with the stimulus type (L, G, or C). The effect of a stimulus is illustrated with a dashed line showing either a horizontal or vertical shift of an agent’s change of condition as the result of the stimulus. For each agent, the simulation was repeated 100 times to obtain the average (thick lines), minimal, and maximal money-gain trajectories.

Examining Fig. 4a, the general trend indicates that high information agents gain money while low information agents lose money. The information is a determining factor and agents need to reach the top of the crowd on information scale before they can consistently gain money. According to the average trajectories, agents at 80% or above on information scale will gain money; agents at 60% on information scale typically maintain their money; and agents at 30% or lower on information scale usually lose money. The similarities between the C and G agents demonstrates that the initial money does not have a significant role in determining the success of an agent. As an interesting anecdote, the lottery agents did not perform well. They lost money and moved toward the lower part of the crescent distribution. The maximum and minimum trajectories give another interesting story showing that pure luck can cause an agent to be very successful or unsuccessful regardless of their money and information status. Altogether, the simulation results point toward an important condition to encourage success: high information is required to improve odds of success. This provides a qualitative answer to what social policy makers and venture capitalists would be interested in, given the constraints of this model.

Since an agent’s evolution pathway also contains significant uncertainty, it would be beneficial to computationally study the characteristics of certain trajectories such as those of agents gaining, maintaining, and losing money to obtain quantitative assessments on what are important to success and the probabilities of the interested pathways. In this exploration, a single pre-lottery-winning agent was simulated 2,000 times with the identical initial parameters. To study the long-term behavior, each simulation ran for 5,000 time-steps. The results show a boomerang distribution plotted with green dots to the right of the crescent distribution of the population background (Fig. 4b). The average trajectories of upper, middle, and lower performing agents are shown in red pathways. As clearly illustrated, starting from the same initial condition, an agent can end with a wide spread of possible states from losing all its money to becoming significantly richer.

The results indicate that information is crucial to an agent’s money status. When an agent’s information gain is low, the agent is very likely to lose money and stay at the lower class. Even to maintain its class, an agent need to gain moderate amount information to surpass 60% or more of the population on information. When an agent gains more information to surpass 80% or more of the population, the agent is likely to move into the upper class on money distribution.

This outcome is based on artificial assumptions but can provide interesting implications to real problems such as class stratification and solidification. While the model does not exactly match the real-world economy, it does suggest that information entities, such as education and fair opportunities to gain and share knowledge, could play a key role in helping middle and lower classes move upward. Understanding this mechanism and its related factors in a real economy would be extremely valuable to policy makers, and can have a profound impact to a society’s wellbeing.

### Model 3: Stock Market

Artificial stock market ABMs examine the price and trade volume of stock and some early models were able to reproduce actual stock market features, including volatility clustering, continual and unpredictable stock price volatility, high skew and kurtosis in investors’ profits distribution^42–45^. However, most models focus on reproduction of only a single stylized fact and were unable to demonstrate more at the same time^29^. Also, these early models are simple in concept but are structurally complicated, which, “contain many deep parameters controlling evolution and learning for which we have only very weak notions of what their values should be”^42^.

To improve the early models, some approaches used neural networks for their rule definitions, which implicitly incorporated some of the information space characteristics^21,45^. However, the weights defining the neural networks were often predetermined and even when weights in the networks were allowed to adapt, the information space interactions and evolution were not explicitly defined and explored.

More recent advancement started to include variables similar to the information space measures in the AIM framework. For example, Ponta *et al*.^29–31^ applied an information variable in the form of sentiments about stocks to single and multi-asset versions of the Genoa Artificial Stock Market^20^. In their models, each agent has its own measure of sentiment towards the market, which is affected by the market trend and connected neighbor agents in their network. These models demonstrated the validity of utilizing an information space, and were then able to reproduce the main univariate stylized facts of markets under a single framework, rather than needing different models for each stylized fact as previously required.

However, these models do not explicitly treat information as a separate space, instead information was handled as a property of each agent. Although mechanically this leads to functions similar to simple AIM models, the conceptual design is different from the AIM’s dual-space framework. Without a clear separation into the dual-space, the rules determining the sentiment are fixed with the agents, which makes the sentiment acting as a dynamic collective consensus of the market’s performance. As such, it limits the freedom of dramatic changes in the information measures, which would be possible with a fully developed independent information space.

For the AIM stock market model shown in this study, the information space contains multiple variables including normal information, divergent information, time-horizon history of the market, historical and recent averages, and market views from a “news” agent. The “news” agent is completely independent of all trading agents. The normal information variable defines a trading agent’s thoughts on when to keep with the status quo, while the divergent information contributes to the likelihood a trading agent’s decision to depart from the status quo. To make decisions, trading agents consider all the information variables. The time-horizon helps decide if an agent thinks the current market trend is good or bad and the “news” agent may affect all trading agents’ information variables by spreading its own market views (SI-S4.4).

**Figure 5:**
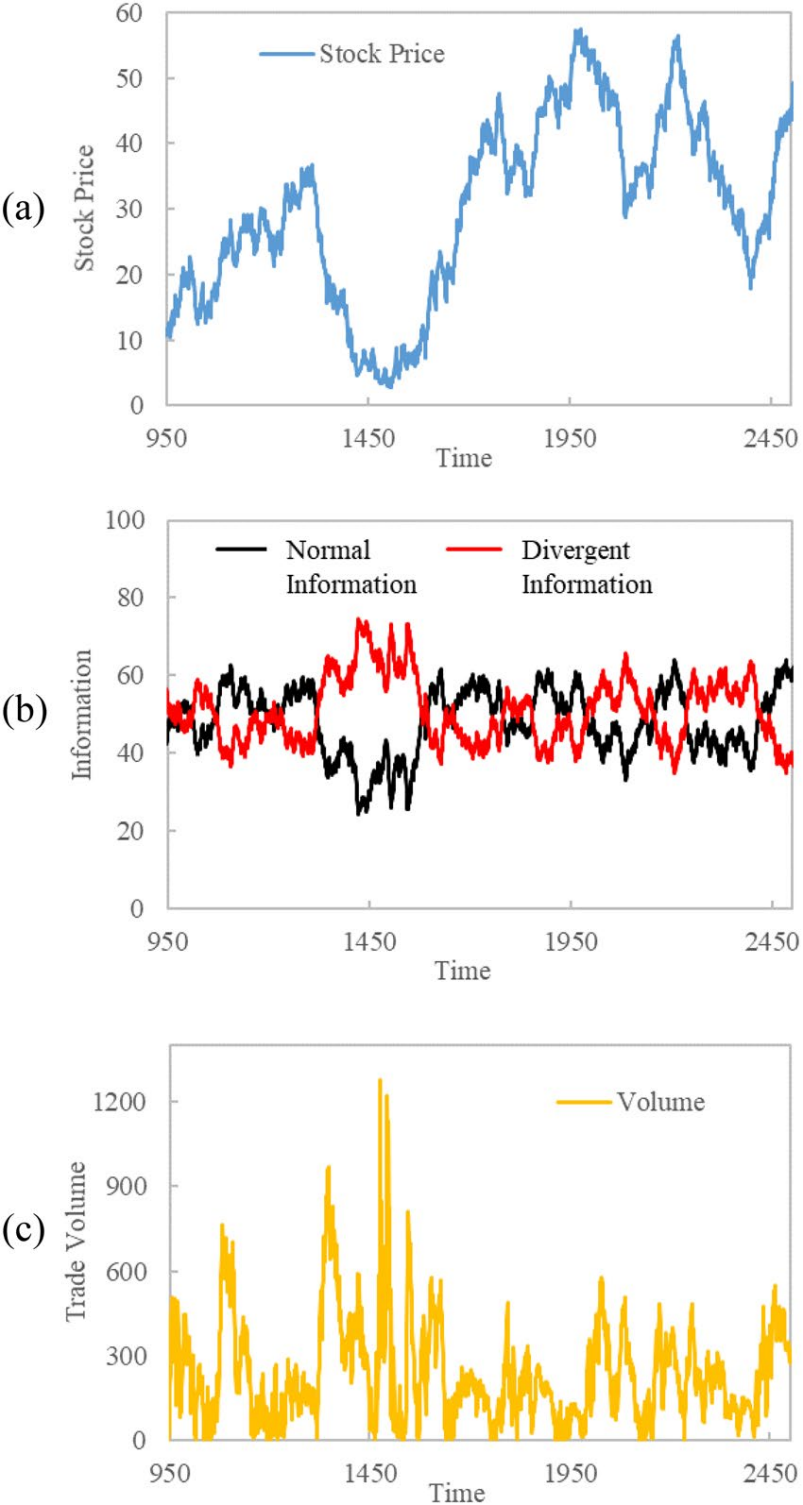
An example run of the stock market AIM simulation demonstrating a significant crash and recovery. (**a**) Stock price, (**b**) normalized normal information and divergent information, (**c**) volume of stock traded.

The behavior of the system can vary greatly from run to run, including market fluctuation, stabilization, and oscillation about an average stock price. However, some of the most interesting behaviors to examine involve more erratic changes that mimic the real-life volatility of actual stock markets. For example, the emergence of a stock collapse is demonstrated in Fig. 5. The normal information and divergent information are normalized with the sum of the two equal to 100. The actual total information in the simulation is always positive and increasing. Periods of high normal information typically occur alongside increasing stock price (time-steps 1100–1300 in Fig. 5) and periods of low normal information occur alongside inferior performance of stock price (time-steps 1350–1550 in Fig. 5). Extrema in either information measure typically coincide with extrema in the volume of stock traded.

Between time-steps 1300–1450, a severe collapse occurs. First the stock price begins to decline which causes a buildup of divergent information. This is caused by agents attempting to profit by selling stock above their historical average and by random fluctuations in demand. Then, the divergent information causes more agents to sell their stock leading to an increase in trade volume (Fig. 5c). The abundance of agents selling stock completes a negative feedback loop as the stock price declines further.

As the stock price decreases, agents begin to become interested in buying the stock again based on their normal information consensus. When the stock’s current price becomes significantly below its recent and historical averages, the condition influences the agents’ decisions to favor buying, which provides a “restoring force” to stabilize the market. As the market stabilizes, the normal information also increases, which weakens the prevalence of the divergent information. Once the normal information increases enough to overcome the effect of the divergent information, the demand will increase and the stock price will start a rising trend.

The AIM model allows this result to emerge from a system of agents reacting to the stock market based on typical buying and selling strategies. However, how the agents utilize these strategies is governed by the information space. The emergence of the collapse and recovery occurs due to the interactions between the information and material spaces without the need to add additional rules. This example demonstrates that by adding the information space, AIMs with a small number of variables and simple rules can simulate emergent radical behaviors in both portions of the dual-space. In addition, using the information space during analysis aids in demonstrating qualitative features of the market.

Note that this model was not analyzed with respect to stylized facts of a real market because the paper focuses on the conceptual AIM rather than real-world accuracy of the model. Additional tuning may be necessary to match with a real-world economy, but that is beyond the scope of this paper.

### Summary

To advance the understanding of information, this study proposes to clearly define three categories of information including local information, external information, and system-state information that each represents a part of the information space. Local information describes features of internal interactions in a system. External information represents the information encoded with a system’s stable states but used for entities external to the system. System-state information takes a statistical approach to describe features of the system at the system-wide level.

To apply information in modeling complex systems, a distinction between the information space and the material space is proposed and applied to develop dual-space AIM models that can more efficiently study intelligent systems. AIM models improve upon many existing ABMs through the addition of the information space, which allows complex behaviors to emerge as the natural outcomes of the system’s evolution without the need to add new rules. This provides greater flexibility and applicability to model many systems with only minor adjustments. Furthermore, the dual-space approach can also be applied to analyze and represent complex interactions between the information and material spaces. The simulation results demonstrate the potentials of using AIM to study a wide variety of social-economic systems.

## Electronic supplementary material

Supplementary Information
