# Supplementary material for: Information of Complex Systems and Applications in Agent Based Modeling
Source: Sci Rep. 2018 Apr 18;8:6177. doi: 10.1038/s41598-018-24570-1 (PMC5906584; doi:10.1038/s41598-018-24570-1)
Supplement: Supplementary file 1 — Supplementary Information [file 41598_2018_24570_MOESM1_ESM.pdf]

Information of Complex Systems and Applications in Agent Based Modeling

Lei Bao & Joseph C. Fritchman

Department of Physics, The Ohio State University, Columbus, Ohio, USA.

# Supplementary Information

## S1. Theoretical system of collisions

### S1.1. Simple collision model

In any elastic collision, the energy and momentum are conserved. However, the total speed ($v_T = \sum|v_i|$) is not necessarily conserved and increases based on the total energy and number of particles in a system. For example, consider a 2D elastic collision between two identical particles (solid hard disks) on a flat frictionless horizontal surface (Figure S1). Before the collision, particle 1 is moving at a constant velocity $\vec{v}_1 = v_0\hat{x}$ and particle 2 is stationary. If particle 1 collides with particle 2 in such a way that the trajectory of particle 1 is altered by 45° above $\hat{x}$, then, solving the problem through energy and momentum conservation, particle 2 must be traveling 45° below $\hat{x}$ with a speed identical to particle 1 as shown in Figure S1. The total kinetic energy is conserved and equally split between the two particles and the total momentum (vector) is also conserved.

This is a simple introductory physics 2D collision problem with an easily calculated result. Typically, the focus is on the fact that energy and momentum are conserved leading to the equations:

$$\frac{1}{2}mv_0^2 = \frac{1}{2}mv_1^2 + \frac{1}{2}mv_2^2 \qquad (S1)$$

$$m\overrightarrow{v_0} = m\overrightarrow{v_1} + m\overrightarrow{v_2} \qquad (S2)$$

which result in:

$$\begin{aligned} |v_1| = |v_2| &= \frac{\sqrt{2}}{2}v_0 \\ v_1 &= \frac{v_0}{2}\hat{x} + \frac{v_0}{2}\hat{y} \\ v_2 &= \frac{v_0}{2}\hat{x} - \frac{v_0}{2}\hat{y} \end{aligned} \qquad (S3)$$

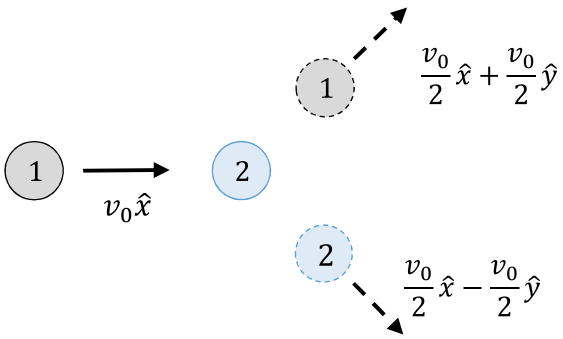


**Figure S1.** Discs are used to represent 2D particles, but will not collide until the centers are in the same location.

The total energy and momentum are both conserved ( $\frac{1}{2}mv_0^2$ and $mv_0$, respectively). However, if the total speed is considered:

$$v_T = \sum |v_i| \qquad (S4)$$

$$v_T = \sqrt{2}\, v_0 \qquad (S5)$$

The total speed has increased! Will the speed continue to increase if more collisions are possible? Two additional discs are added to the setup in a configuration such that the discs leaving the first collision will each collide with one of the new discs and their paths will again change by 45°. Starting from the result of the first collision, momentum and energy still must be conserved in the 2nd set of collisions and the following velocities are calculated:

$$v_1 = \frac{v_0}{2}\hat{y} \qquad v_2 = \frac{v_0}{2}\hat{x} \qquad v_3 = \frac{v_0}{2}\hat{x} \qquad v_4 = -\frac{v_0}{2}\hat{y}$$

$$v_T = 2v_0 \qquad (S6)$$

The total speed again increases by a factor of $\sqrt{2}$. This pattern can continue if there are new stationary discs for each moving disc to collide with. In this situation (with $n_c$ defined as the number of sets of collisions):

$$v_T(n_c) = v_0(2)^{\frac{n_c}{2}} \qquad (S7)$$

As a first approximation, this example demonstrates that even though the total energy and momentum are held constant, the total speed increases exponentially! This is due to the energy being dispersed through an increasingly larger number of discs.

However, this system is not physical for several reasons:

- It does not consider collisions between moving discs (fails at 4th collision set)
- It only considers collisions between two discs (fails at 4th collision set)
- It assumes new discs will be readily available to collide with (fails by the 9th collision set)
- It only considers 2D motion (uses discs instead of hard spheres)

To rectify these issues, an event-driven simulation may be employed to track every particle and collision. Removing the constraints from this example leads to an ideal gas model. To begin with, a 2D ideal gas will be examined.

### S1.2. Theoretical $p_T$ derivation

The total absolute momentum, $p_T$ is defined as the sum of the magnitude of momenta of all $N$ particles in a system.

$$p_T = \sum_{i=1}^{N} p_i \qquad (S8)$$

To theoretically find $p_T$, the 2-dimensional equilibrium speed distribution for classical particles in an isolated box is a simple gamma distribution—the Maxwell-Boltzmann distribution:

$$f(v) = 2\pi v f(v) = 2\gamma v e^{-\gamma v^2} \qquad (S9)$$

Converting this to the momentum distribution ($\alpha = \beta/2\text{m}$):

$$f(p) = 2\,\alpha\,p\,e^{-\alpha p^2} \tag{S10}$$

From the distribution, characteristics of particles in the gas can be found. The most probable speed can be calculated by differentiating the distribution function to find the maximum:

$$\frac{df(p)}{dp} = 0 \tag{S11}$$

$$p_p = \sqrt{\frac{1}{2\alpha}} = \sqrt{mk_bT} \tag{S12}$$

The average speed can be calculated by integrating:

$$\int_0^\infty f(p)p\,dp = \langle p\rangle \tag{S13}$$

$$\langle p\rangle = \frac{1}{2}\sqrt{\frac{\pi}{\alpha}} = \sqrt{\frac{m\pi}{2\beta}} \tag{S14}$$

Similarly, the average of the speed squared can be found by integrating:

$$\langle p^2\rangle = \int_0^\infty f(p)\,p^2\,dp \tag{S15}$$

$$\langle p^2\rangle = \frac{1}{\alpha} = 2mk_bT = \frac{4}{\pi}\,\langle p\rangle^2 \tag{S16}$$

The root mean square speed is then:

$$p_{rms} = \sqrt{\frac{\Sigma p_i^2}{N}} = \sqrt{\langle p^2\rangle} \tag{S17}$$

$$p_{rms} = \sqrt{2mk_bT} \tag{S18}$$

The most probable speed and the root mean square speed are both proportional to the average speed:

$$\sqrt{\frac{\pi}{2}}\,p_p = \langle p\rangle = \sqrt{\frac{\pi}{4}}p_{rms} \tag{S19}$$

The total energy in the system is conserved such that the initial kinetic energy must equal the equilibrium kinetic energy.

$$\frac{p_0^2}{2m_0} = \Sigma\left(\frac{p_i^2}{2m_i}\right) = \frac{1}{2m}\,N\,\langle p^2\rangle = \frac{1}{2m}\,N\frac{4}{\pi}\,\langle p\rangle^2 \tag{S20}$$

Which leads to a relationship between the average absolute momentum and the initial momentum.

$$\langle p \rangle = \sqrt{\frac{\pi}{4}} \frac{p_0}{\sqrt{N}} \tag{S21}$$

Total absolute momentum is then the average absolute momentum multiplied by the number of particles. This leads to the total absolute momentum being directly dependent to the square root of N times the starting momentum $p_0$:

$$p_T = N\langle p \rangle = \sqrt{\frac{\pi}{4}} p_0 \sqrt{N} \tag{S22}$$

### S1.3. Calculating $p_T$ for a system of particles of different masses

Above, all derivations assumed each particle had the same mass, $m$. Here, the case of different masses will be explored. First, the total energy, total number of particles, average energy of particles, and temperature will remain the same. If there are $n$ different masses of particles in the system, then the total number of particles in the system is:

$$N = \sum_{i=1}^{n} N_i \tag{S23}$$

Where $N_i$ is the number of particles with mass $m_i$. The total kinetic energy remains the same due to the incoming particle (with $p_{ij}$ as the momentum of the $j^{th}$ particle with mass $m_i$):

$$\begin{aligned} E_T = \frac{p_0^2}{2m_0} &= \sum_{i=1}^{N} \left( \frac{p_i^2}{2m_i} \right) \\ &= \sum_{i=1}^{n} \sum_{j}^{N_i} \left( \frac{p_{ij}^2}{2m_i} \right) \\ &= \sum_{i=1}^{n} N_i \frac{\langle p_i^2 \rangle}{2m_i} \end{aligned} \tag{S24}$$

Also, the average energy per particle remains the same:

$$E_{avg} = \frac{E_{tot}}{N} = \frac{\langle p_i^2 \rangle}{2m_i} \tag{S25}$$

for $i = 1 \; to \; n$. This leads to the average total energy for each mass of particle being:

$$E_i = \frac{N_i}{N} E_T \tag{S26}$$

And the average energy in the previous derivations can be replaced by this energy to find distributions for each mass of particle. This is equivalent to adjusting the constant $\alpha_i = \frac{m_0}{m_i} \alpha$

$$f_i(p) = 2\, \alpha_i \, p_i \, e^{-\alpha_i p_i^2} \tag{S27}$$

Then the previous results hold as well, shifting $\alpha \rightarrow \alpha_i$, $m \rightarrow m_i$, and $p \rightarrow p_i$.

$$\langle p_i \rangle = \frac{1}{2}\sqrt{\frac{\pi}{\alpha_i}} \qquad (S28)$$

$$\langle p_i^2 \rangle = \frac{1}{\alpha_i} = \frac{4}{\pi}\langle p_i \rangle^2 \qquad (S29)$$

$$\frac{N_i}{N}\frac{p_0^2}{2m_0} = \sum\left(\frac{p_i^2}{2m_i}\right) = \frac{1}{2m_i} N_i \langle p_i^2 \rangle = \frac{2N_i}{\pi\, m_i}\langle p_i \rangle^2 \qquad (S30)$$

$$\langle p_i \rangle = \sqrt{\frac{m_i}{m_0}}\sqrt{\frac{\pi}{4}}\frac{p_0}{\sqrt{N}} = \sqrt{\frac{m_i}{m_0}}\langle p_0 \rangle \qquad (S31)$$

The average momentum for each particle is former average momentum multiplied by the square root of the ratio of the particle's mass and the original particle's mass.

$$p_T = p_0\sqrt{\frac{\pi}{4}}\left(\sum \frac{N_i}{\sqrt{N}}\sqrt{\frac{m_i}{m_0}}\right) \qquad (S32)$$

### S1.4. Adding energy incrementally

Adding energy to the system incrementally instead of all at once does not change to equilibrium solution. Calculations so far have assumed that all the energy was given at one time to a frozen system. Instead, now the case of adding $N_E$ packets of energy to the system (such that $\frac{1}{2}mv_0^2 = \sum_i^{N_E}(\frac{1}{2}mv_i^2)$ *and* $m\vec{v}_0 = \sum_i^{N_E} m\vec{v}_i$). To add the energy, a particle will be added to the system with velocity $\vec{v}_i$ and the system will be allowed to reach equilibrium. The process will then be repeated until all the energy has been deposited. The system will contain enough particles such that the additional particles will cause negligible effects to the system ($N \gg N_E$).

After the first energy packet is added to a frozen system, the format of the total velocity (and other descriptors) will be identical to the prior case:

$$p_T = \sqrt{\frac{\pi}{4}}p_i\sqrt{N} \qquad (S33)$$

Additional particles added to the system result in:

$$E_T = \sum\left(\frac{1}{2}m\, p_i^2\right) = \frac{1}{2}m\, N\, \langle p^2 \rangle = \frac{1}{2}m\, N\frac{4}{\pi}\langle p \rangle^2 \qquad (S34)$$

And the total absolute momentum becoming:

$$p_T = \sqrt{\frac{\pi}{4}}\sqrt{\sum\nolimits_i^{N_E} p_i^2}\,\sqrt{N+N_E} \approx \sqrt{\frac{\pi}{4}}\sqrt{\sum\nolimits_i^{N_E} p_i^2}\,\sqrt{N}\left(1+\frac{1}{2}\frac{N_E}{N}+\cdots\right) \qquad (S35)$$

If the total energy added to the system is equivalent to the energy with of a single particle with momentum $p_0$, then the $p_i^2$ summation is just $p_0^2$:

$$\frac{p_0^2}{2m} = \frac{1}{2}\sum_i^{N_E} \frac{p_i^2}{m} \rightarrow p_0^2 = \sum_i^{N_E} p_i^2 \qquad (S36)$$

This shows that the equilibrium state is unchanged regardless of the energy addition method. If the number of particles is not large compared to the number of particles added, then the adding many energized particles method with $N$ frozen particles must be compared to energizing a single particle in a system with $N + N_E$ frozen particles.

### S1.5. Time constant data

Prior to reaching an equilibrium state, the simulations show that $p_T$ increases in a sigmoidal fashion (Figure **S2**). When very few particles are moving, it is expected that $p_T$ will quickly increase as most collisions will be with stationary particles. When most of the particles are moving, the $p_T$ increase will be minimal because most collisions involve two moving particles. This suggests the form:

$$\frac{dp}{dt} = \frac{p(t)}{C}(C - p(t)) \qquad (S37)$$

The solution to this differential equation is a sigmoid with $C = 2(p_T - p_0)$ and a constant offset of $-(p_T - p_0) + p_0$:

$$p(t) = \frac{2(p_T - p_0)}{1 + e^{-\alpha t}} - (p_T - p_0) + p_0 = \frac{1 - e^{-\alpha t}}{1 + e^{-\alpha t}}(p_T - p_0) + p_0 \qquad (S38)$$

The constant $\alpha$ will depend on parameters of the system including number of particles, particle size, energy, and size of box.

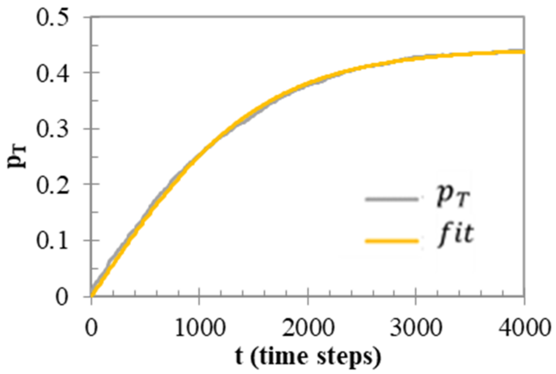


**Figure S2.** $p_T$ and its sigmoid fit vs time for simulation ($N = 2500, v_0 = 0.01$).

This sigmoid shape holds for all simulations run (example in Figure S2) and the time it takes for the system to reach 95% of the maximum $p_T$ forms a power law relation. For most cases, it follows $t_c = A\, N^{-0.5}$ as shown in Figure 10 a. This does not fit as well for the higher velocity ( $v_0 = 10$ ) case. The constant $A$ fits the form $A = \gamma\, p_0^{-1.5}$. This corresponds to $\alpha = \frac{\ln(39)}{\gamma}\sqrt{N p_0^3}$ for the constant in the sigmoid fit. From the fit in Figure S3b, $\gamma \approx 2{,}000$. The final equation is then (with $\alpha_0 = \frac{\ln(39)}{\gamma} \approx 1.8\, \times 10^{-3}$):

$$p(t) = \frac{2(p_T - p_0)}{1 + e^{-\alpha t}} - p_T + 2p_0 = \left[\sqrt{\frac{\pi}{4}} \frac{1 - e^{-\alpha_0 \sqrt{N p_0^3}\, t}}{1 + e^{-\alpha_0 \sqrt{N p_0^3}\, t}} \left(\sqrt{N} - \sqrt{\frac{4}{\pi}}\right) + 1\right] p_0 \qquad (S39)$$

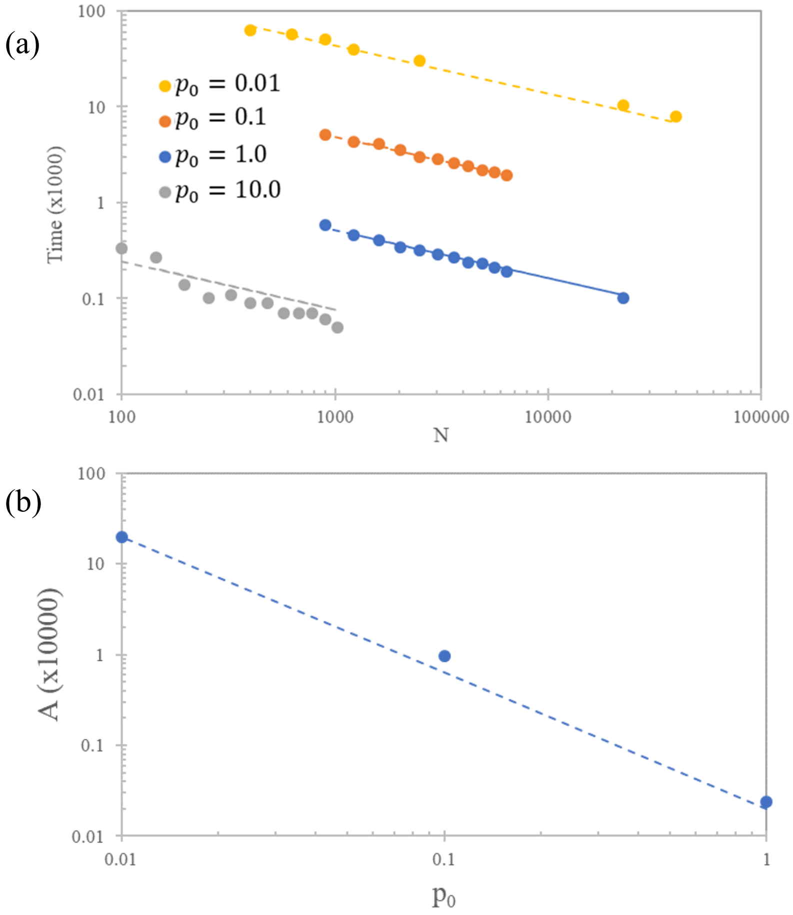


**Figure S3**. Equilibrium plots. (a) Time to reach equilibrium, (b) Coefficient A.

## S2. Entropy calculations

### S2.1. Entropy theory

Since $p_T$ is created as the result of interactions (collisions) between individual particles, it then must represent features of such interactions. From the initial to the equilibrium situation, the particles in the system increase their mobility (as reflected by $p_T$) and the system becomes more interactive and disordered as individual particles are moving in all possible directions and with a wide range of momentum values. The first natural connection is to compare $p_T$ and entropy, which is defined to describe the scale of disorder of a system.

Entropy is a common topic of confusion for both novice and experts alike [1,2,3,4]. Part of the confusion is due to Boltzmann's description of large entropy as disordered and later followed by Helmoholtz, Gibbs, and countless textbook authors [5]. Chemistry texts, and others, have described

entropy as, "a measure of energy unavailable to do work." While the expert may understand the spirit of this definition, the novice will quickly confuse and mix the energy and entropy concepts.

In addition, multiple different types of entropy exist such as the experimental form given by Clausius, the Gibbs entropy, Boltzmann entropy, and Shannon entropy, which will be briefly reviewed below. The entropy of a system has been defined both classically and statistically. For a classical system, the change in entropy is defined following from the Second Law of Thermodynamics and Clausius' Thereom [6], which lead to the definition of entropy as a state function and the macroscopic definition of entropy. The maximum Gibbs' entropy, which occurs when all states are equally likely, is equivalent to Clausius' classical definition of entropy. In a classical system, the energy distribution is continuous and differential entropy will be needed, which will be shown below [7,8,9,10].

First, revisiting the *N*-particle collision problem, the system's initial entropy with only one particle moving at $p_0$ and $(N-1)$ particles stationary is (for very large $N$):

$$\lim_{N\to\infty} S_0 = k_b \ln N \qquad (S40)$$

The system's entropy then increases through collisions and reaches an equilibrium maximum. The equilibrium entropy varies with $p_0$ and $N$, and has a similar increasing trend as the total absolute momentum $p_T$ (shown below).

Differential entropy allows further calculation of entropy in a classical system with a continuous energy distribution. Regular differential entropy as described by Shannon and Weaver 7 will result in a natural log of the momentum. This develops a new problem: taking the log of a variable with units is not allowable and this entropy is then not invariant under a change of variables. To counteract these issues, Jayne introduces the limiting density of discrete points adjustment [8,9,10]. The result is an added constant $\Delta$ with momentum units (in this case) to keep the logarithm unitless and discretize the distribution:

$$S \to -Nk_b \int_{-\infty}^{\infty} f(p)\ln(f(p)\Delta)\, dp \qquad (S41)$$

This can be integrated analytically for the ideal gas distribution [11] ($\gamma$ is the Euler-Mascheroni constant):

$$S_{2D} = N\left[\ln\left(\frac{p_0}{\Delta}\right) - \frac{1}{2}\ln(N) + \ln(2) + \frac{\gamma}{2} + 2\right] \qquad (S42)$$

Similar results can be achieved by applying a statistical approach to the entropy derivation, but will not be shown here. Combining the natural logarithms for the 2D entropy equations leads to an equation of the form (with C as constants):

$$S_{2D} = N\left(\ln\left(\frac{p_0}{\Delta\sqrt{N}}\right) + C\right) \qquad (S43)$$

Naively applying this equation would allow for peculiar behavior of the entropy at large $N$ and small $p_0$ because it is possible that $\frac{p_0}{\Delta\sqrt{N}} < 1$. To sidestep this pitfall, it is important to remember that the distribution was discretized in order to carry out the integration. The $\Delta$ is the bin size used in this discretization. It must be adjusted depending on $p_0$ and $N$ to ensure that the shape of the distribution remains the same. A sufficiently small $\Delta$ will work for a wide range of possible systems and yields the expected entropy behavior; entropy rises when energy or

particles is added to the system (increasing $p_0$ or $N$) and tends to zero when the system is frozen at absolute zero $\left(\Delta \sim \frac{\sqrt{N}}{p_0}\right)$.

Because the choice of Δ can be subjective, it is beneficial to look primarily at the change in entropy between two non-frozen states:

$$\Delta S_{2D} = k_b \left[ -\frac{1}{2} \ln\left(\frac{N_2^{N_2}}{N_1^{N_1}}\right) + \ln\left(\frac{{p_0}_2^{N_2}}{{p_0}_1^{N_1}}\right) + (N_2 - N_1)(C - \ln(\Delta)) \right] \qquad (S44)$$

When the number of particles is held constant, a familiar equation is recovered:

$$(\Delta S_{2D})_N = k_b N ln\left(\frac{p_{0_2}}{p_{0_1}}\right) = k_b N ln\left(\frac{\langle p\rangle_2}{\langle p\rangle_1}\right) = k_b N ln\left(\frac{T_2}{T_1}\right) \qquad (S45)$$

This is the change in entropy caused by a change to the systems internal energy as calculated using the first law of thermodynamics, demonstrating consistency between classical and statistical entropy. If the number of particles is not constant, then the choice of Δ will play a role.

### S2.2. Collisions rate calculation

One feature of ideal gases and these simulations is that the equilibrium rate of collisions is predictable. While the energy is dispersing throughout the system, the rate of collisions increases linearly before plateauing (Figure S4).

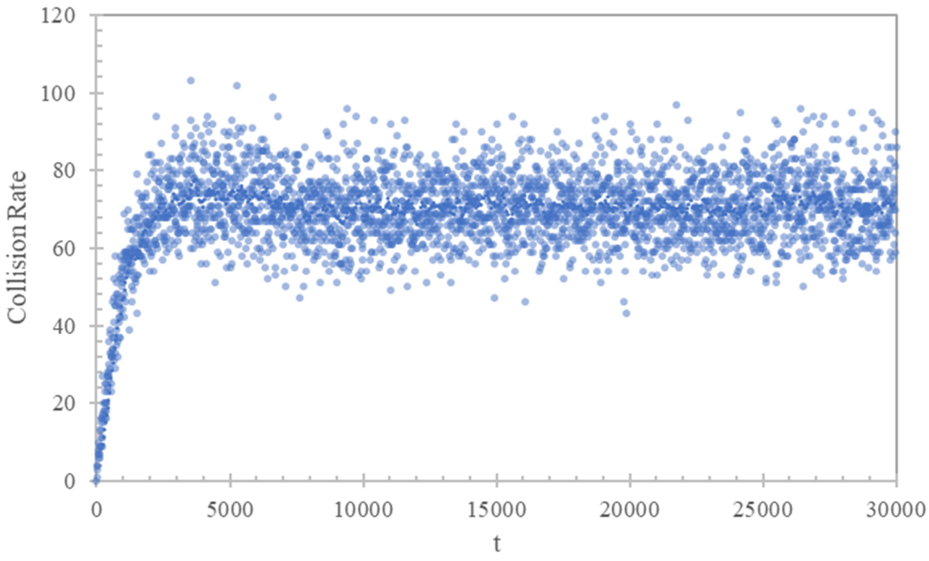


**Figure S4.** Collision rate vs. time step ($N = 4225, p_0 = 0.01$).

The average rate of collisions in the plateau region can be calculated by finding the mean free path for particles in the system. To do so, it is beneficial to look at the number of collisions that would occur if a particle moved through a region with velocity $\vec{v}$. Initially an assumption is made such that only one particle is moving. The average number of collisions can then be calculated as the area swept by a moving particle multiplied by the number density of particles (using the root mean square velocity: $p_{rms} = \sqrt{\langle p^2\rangle}$ ):

$$n_c = 2d\frac{\langle p\rangle}{m}\,\Delta t\, n_A \qquad (S46)$$

However, in a system that has reached equilibrium, all particles may be moving. To account for this, the average relative momentum of particles is needed. This, in turn, is then related to the average absolute momentum of particles and the number of collisions can be revised:

$$\langle p_{rel}\rangle = \sqrt{2}\langle p\rangle \qquad (S47)$$

$$n_c = 2d\sqrt{2}\frac{\langle p\rangle}{m}\,\Delta t\, n_A \qquad (S48)$$

The mean free path is then the distance traveled by the particle divided by the number the number of collisions occurring:

$$l = \frac{\langle p\rangle \Delta t}{2\, d\,\sqrt{2}\,\langle p\rangle \Delta t\, n_A} \qquad (S49)$$

$$l = \frac{a^2}{N\, d\,\sqrt{8}} \qquad (S50)$$

The average time in between collisions for a single particle is:

$$\tau = \frac{l}{\frac{\langle p\rangle}{m}} \qquad (S51)$$

$$\tau = \frac{m\, a^2}{d\, p_0}\sqrt{\frac{1}{2\,\pi\, N}} \qquad (S52)$$

The frequency of collisions for a single particle is

$$\bar{f} = \frac{1}{\tau} \qquad (S53)$$

The total frequency of collisions is (subscript p refers to particle-particle collisions)

$$F_p = \sum\nolimits_{i=0}^{N} f_i = \frac{1}{2}N\bar{f} = \frac{N\langle p\rangle}{2\, l\, m} \qquad (S54)$$

(Note that the factor of ½ corrects for double counting collisions in the sum)

$$F_p = \frac{\sqrt{\pi}}{a^2\sqrt{2}}d\frac{p_0}{m}\,N^{\frac{3}{2}} = \frac{2R\sqrt{2}}{a^2}\,N\frac{p_T}{m} \qquad (S55)$$

Collisions with the walls can be directly related to the pressure in the box. From the ideal gas law for 2D:

$$P = \frac{Nk_bT}{A} \qquad (S56)$$

Elastic collisions with the wall apply an average impulse

$$\langle I\rangle = \frac{\Delta p}{\Delta t} = \frac{2m\langle v\rangle}{\Delta t} = \frac{P}{F_w}L \qquad (S57)$$

With $F_w$ defined as the total number of collisions with all walls per unit time. L is the total length of the walls ($A = 4a$). Solving the equations for $F_w$ and substituting in the average velocity for $k_b T$ leads to:

$$F_w = \frac{2}{a\sqrt{\pi}}\frac{p_0}{m}\sqrt{N} = \frac{4}{\pi a}\frac{p_T}{m} \tag{S58}$$

However, the wall collision rate is linearly proportional to $p_T$with no $N$ dependence because wall collisions do not change $p_T$ of the system. Instead they only redirect a particle, keeping speeds constant. Wall collision rate is therefore only a measure of $p_T$ (and pressure) as opposed to a determining factor.

In addition, the collision rate follows a sigmoidal pattern over time, similar to $p_T$ (Figure S2, Figure S4). This suggests that the collision rate as a function of $p_T$ should apply even during the transient period:

$$F_p(t) = \frac{2R\sqrt{2}}{a^2} N \frac{p_T(t)}{m} \tag{S59}$$

### S2.3. Comparing Entropy, Total Momentum, and Collision rate

The remarkable thing about the collision rate is that it can be directly related to several measurable quantities including pressure and temperature, and $N \ln F$ is related to the entropy! The log of the collisions occurring in time $\Delta t$ with the walls (or other particles) can be related to the entropy (if $\Delta = am/\Delta t$):

$$\ln(F_p \Delta t) = \ln\left(\frac{p_0}{\left(\frac{am}{\Delta t}\right)}\right) + \frac{3}{2}\ln(N) + \ln\left(\frac{2R}{a}\sqrt{\frac{\pi}{2}}\right) \tag{S60}$$

$$\frac{S_{2D}}{N} = \ln(F_p \Delta t) - 2\ln(N) - \ln\left(\frac{2R}{a}\sqrt{\frac{\pi}{2}}\right) + \ln(2) + \frac{\gamma}{2} + 2 \tag{S61}$$

$$F_{pp} = \frac{N^2 e^{S_{2D}/N} e^{c_{pp}}}{\Delta t} \tag{S62}$$

Letting $c_{pp} = \ln(2) - \ln\left(\frac{2R}{a}\sqrt{\frac{\pi}{2}}\right) + \frac{\gamma}{2} + 2$ in eq. S61. The collision rate is able to describe both macro- and microscopic behavior of a system. At equilibrium, no additional $p_T$ (on average) is created through these collisions. Prior to equilibrium, most collisions will create additional $p_T$.

With relations between the Entropy, $p_T$, and particle collision rates, the three can be compared to examine the usefulness in describing systems (Figure S5). The $p_T$ is the simplest relation: linear with $p_T$ and proportional to $\sqrt{N}$. This offers a way to describe changes to the system based on changing numbers of particles or energy gains/losses. The collision rate is linearly proportional to $p_T$, but expands as $N^{3/2}$. However, the collision rate will have an upper bound for $N$, when no more particles can fit inside the system:

$$N < \left(\frac{a}{2R}\right)^2 \tag{S63}$$

Among the three variables, $p_T$ and $f_p$ are directly connected to local interactions. That is, $p_T$ and $f_p$ can both take a bottom-up approach. Using the information of individual particles' momenta and collision rates, one can accumulate these to produce $p_T$ and $f_p$ of a local region of an unknown system or the entire system that may be constantly changing. As a result, the total absolute momentum and collision rate are measures directly reflecting information of local interactions. These local region interactions are of great interest in large-N interactive systems such as bio-chemical reactions and social dynamics, whose local level interactions can accumulatively contribute to the total group behavior.

In contrast, the entropy gives a measure of the degree of disorder of a system based on a macroscopically defined probabilistic framework. That is, to determine the probability distribution of states of the system, the entire system and its possible states need to be known. This reflects a top-down process that begins with defining the entire macroscopic system and down to the probabilities of specific states determined based on the macroscopic system. For the entropy measure, a bottom-up approach is impossible. The information of individual particles of a system cannot be used to calculate the entropy without knowing the complete probabilistic configuration of the entire system. Therefore, the entropy is a macroscopic measure of the system that doesn't directly respond to local level interactions.

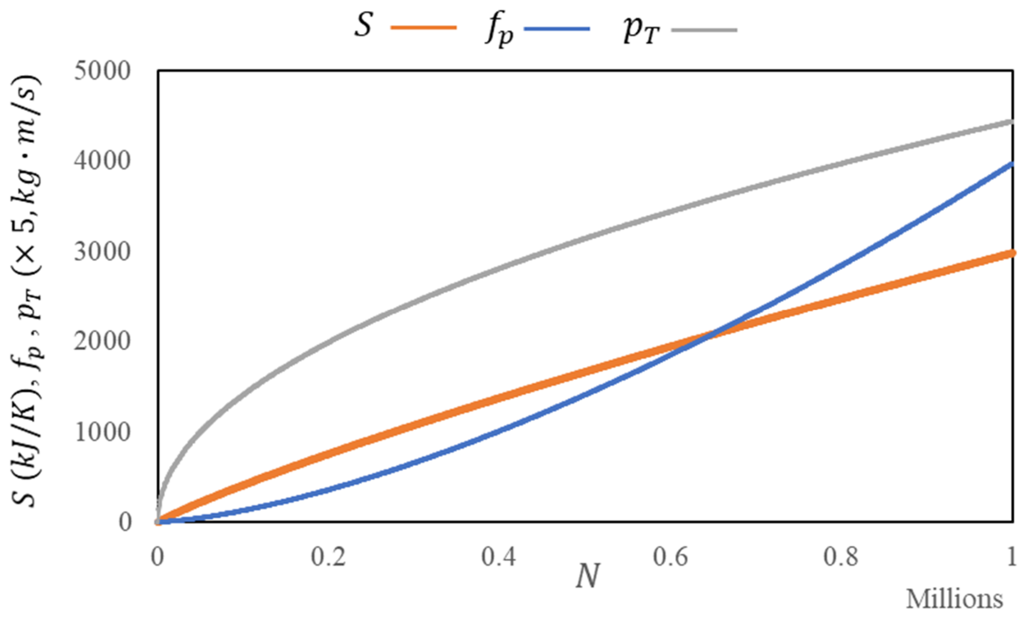


**Figure S5.** Entropy, $p_T$, and particle collision rate comparison.

### S3. 3D Calculations

The above derivations were performed assuming a two-dimensional system. Similar derivations lead to three-dimensional analogs:

Total absolute momentum

$$p_{T_{3D}} = N\langle v\rangle = \sqrt{\frac{8}{3\,\pi}}p_0\sqrt{N} \quad (S64)$$

Differential entropy

$$S_{3D} = N\left[\ln\left(\frac{p_0}{\Delta}\right) - \frac{1}{2}\ln(N) + \ln\left(\frac{1}{2}\sqrt{\frac{\pi}{6}}\right) + \gamma - \frac{1}{2}\right] \quad (S65)$$

Collision rate

$$F_{p_{3D}} = \frac{2}{a^3}\sqrt{\frac{\pi}{3}}d^2\frac{v_0}{m}N^{\frac{3}{2}} = \frac{4R^2\pi}{a^3\sqrt{2}}N\frac{p_T(t)}{m} \quad (S66)$$

## S4. Agent and Information Based Modeling (AIM)

### S4.1. Three types of information

Local, system-state, and external information were all discussed in the main paper. To help explain these three types of information, imagine a flashlight turning on and off to send a Morse code signal. The interactions of electrons in the wire contain the local information. The state of the system (on or off) is the system-state information. Then the external information is the message being transmitted through Morse code.

A similar parallel can be drawn to information encoded in magnetic tape. Local information is in magnetic polarization of local domains on the tape. The state of each bit (0 or 1) is the system-state information. Then the external information is the message encoded with the sequences of 0's and 1's.

### S4.2. General AIM

AIM models can be summarized as a recursive system of equations spanning the information-material dual-space. The information and material spaces are separate dimensions and may evolve completely independently from each other. However, the most interesting cases allow interaction between the two spaces while still allowing evolution mechanisms in each space to function independently. For example, an agent completes a trade in the material space and gains information relating to that trade such that both its material and information measures increase. Without interacting with the material space, the agent may use this information to improve their abilities to trade, demonstrating an increase in information independent from the material space.

For a system at time $t$, the material space is represented by $M(t)$ and the information is represented by $I(t)$.

$$\begin{aligned} M(t + \Delta t) &= F\big(a\, M(t), b\, I(t)\big) \\ I(t + \Delta t) &= f\big(c\, M(t), d\, I(t)\big) \end{aligned} \quad (S67)$$

Where $F$ and $f$ are system dependent functions and the constants a, b, c, and d are either 0 or 1. Typically, these can be summarized in a few categories. First, information moderates material interactions by deciding which agents can interact and how strong these interactions are. If the information interactions were ignored ($b = 0$), the behavior should be identical to a non-intelligent system. Second, being directly involved in an interaction will lead to information

change as agents learn more about the nature of interactions. Third, information can spontaneously change due to significant events which affect the system and add additional $\delta I$ and $\delta M$ terms above. The rules governing each of these will need defined for each system.

The constants a, b, c, and d allow 16 different systems of equations for evolution. From these 16, only 6 unique patterns exist and are displayed in Table S1. These include: M depending on both I and M, M depending on either only I or only M, only M changes at a non-constant rate, only I changes at a non-constant rate, and both I and M only change at constant rates. Each of these patterns can be related to real world situations, hinting at how to set up a simulation to model these:

1. M depends on I and M: Agents carefully consider their and the system's information and material spaces before acting. These systems will allow individuals to formulate their own plans based on desired goals. Methods of information growth will be system specific. Examples include an economy based on Capitalism.
2. M only looks at M: Agents in this system can only make decisions based on material spaces. The information space is superfluous. Examples include non-intelligent systems such as an ideal gas.
3. M only looks at I: Agents in this system only consider the information space when making decisions. Indirectly they are considering the material space through the information space. Examples include students in high school or university who may not have many material space demands, or wealthy individuals who are able to use their material space without limits.
4. Only M changes: Agents here have an information space, but it does not evolve. Examples include economies where information is strictly controlled, such as under communism.
5. Only I changes: No material exchanges occur. This could represent a purely academic setting with free exchange of ideas.
6. No M or I dependence: This is the simplest system possible. All agents evolve at predetermined rates without regard to the system or individual's details.

**Table S1:** Patterns of interactions in a system containing one material dimension and one information dimension.

| | *a* | *b* | *c* | *d* | |
|---|---|---|---|---|---|
| M depends on I and M | 1 | 1 | 1 | 1 | $M = F(M(t) + I(t))$ |
| | 1 | 1 | 1 | 0 | |
| | 1 | 1 | 0 | 1 | |
| M only looks at M | 1 | 0 | 1 | 1 | $M = F(M(t))$ |
| | 1 | 0 | 1 | 0 | |
| | 1 | 0 | 0 | 1 | |
| M only looks at I, I is dynamic | 0 | 1 | 1 | 1 | $M = f(I(t))$ |
| | 0 | 1 | 1 | 0 | |
| | 0 | 1 | 0 | 1 | |
| Only M changes | 1 | 1 | 0 | 0 | $I = I_0$ |
| | 1 | 0 | 0 | 0 | |
| | 0 | 1 | 0 | 0 | |
| Only I changes | 0 | 0 | 1 | 1 | $M = M_0$ |
| | 0 | 0 | 1 | 0 | |
| | 0 | 0 | 0 | 1 | |
| Constant or constant change | 0 | 0 | 0 | 0 | $M = M_0 \text{ and } I = I_0$ |

Consider a non-intelligent system ($b = 0, d = 0$). Here, the information will be bounded by the material space interactions. This occurs because both the material space and information space are only dependent on the material space:

$$\begin{aligned} M(t + \Delta t) &= F(M(t)) \\ I(t + \Delta t) &= f(M(t)) \end{aligned} \qquad (S68)$$

A non-intelligent system is simply a generalized information model which functions as a material space only model with no information interactions. The information space exists but it is inconsequential in determining the behavior of the system and is often ignored. The elastic collision system discussed previously follows this non-intelligent model with the upper bound on information $I(t) \leq p_{max}$.

Contrasting the non-intelligent model, intelligent systems consist of agents who can learn from interactions and will have unbounded information. This culminates in a system of equations containing functions within functions, which can lead to non-linearity and emergent behavior in both material and information spaces.

The focus of the main paper is on how to use information in a dual-space AIM, how information will affect a model, and how information can affect analysis and representation of the model. In general, the use of information in AIM differs from previous models in two ways:

- AIM treats information as an independent dimension that can evolve in its own space independent of the material space. General ABM models treat information as a component of material space rules rather than an independent dimension.

- AIM uses information not only for modeling but also for analyzing and representing a system's behavior. Previous models use information as a basis for rules, but rarely analyze the evolution of information within the model.

### S4.3. Comparison of AIM to previous uses of information

It is evidential that others have not used information as an independent dimension. Instead, others have used information as a rule construction tool. This natural because information plays a role in all decision making and decision making typically makes use of a ruleset. Typically, in the context of agent-based models, rule construction occurs such that desired actions occur based on available information. For example, "if it is raining, then you should use an umbrella." An agent following this rule while it is raining would use an umbrella. Another agent may follow a similar rule for a raincoat. If the two agents encountered each other, would each think the other was not following the rule? Would an agent attempt to convert the other to its rule? If these rules are hard-coded for these agents, then they have no choice but to follow the rule. In many agent-based models, this is exactly the case—agents are unable to adjust their rules and must act according to pre-defined plans. Furthermore, the rule does not give the underlying reason for needing an umbrella or raincoat—to avoid getting wet. A human would be able to make the logical connection that the rule is designed to prevent one from getting wet, but the agent would be unable to generalize.

In the above approaches, the info is expressed in rules and is not treated as an independent dimension to allow its own evolution. Based on a rule, it is difficult to reversely reconstruct the information measure from the rule as the rule can involve many factors from both the info space and the material space. Therefore, information is used implicitly here in the material space as a component (variable or condition) for rule definition.

This is where the beauty of agent and information based models shines. AIMs seek to expand upon these hard-coded rulesets by allowing an agent to change its rules based on the information available. In the above example, a complete AIM model could result in a new rule, "if you are likely to get wet, then you should use an appropriate piece of gear for the situation." This allows the agent to understand that any situation when one is likely to get wet could require taking an action to prevent this. The reason one might get wet could be caused by a variety or combination of factors: rain, wind, or sprinklers for example. If it is just raining, an umbrella or raincoat could be chosen based on an agent's preference. However, if it is windy and raining, the umbrella may not be a smart choice. In traditional models, this behavior could occur through a series of "if-then" statements. In AIM, this occurs functionally with arrays of weights in a feed-forward neural network. This network stores an agent's preferences within its weights. Through interactions with other agents, the preferences of agents may change. With hard-coded rules, this would require changing an "if-then" statement. In AIM, this occurs through adjusting weights which can be a natural process from the interaction of the information space. Not only does information moderate actions an agent takes, the information space evolves with the agent.

The existing models, which use information, often try to work the effect of information into a traditional non-information based ABM structure (single-dimension), which is mostly static rule based. (tradition or old habit). AIM provides a new structure that has multiple dimensions and allows information to be an evolutionary dimension with unlimited possibility.

**Table S2.** General comparison between information use in AIM and previous models

| Information definition usage | AIM | Previous Info model |
|---|---|---|
| Definition | Local, state-wide, and external | Indiscriminate |
| Independent dimension | Info space can evolve independent of material space | Expressed in static rules. No independent dimension. |
| System | Models intelligent systems that have information dimension evolutions. | Non-intelligent systems that can embed information within material space only. |
| Operation | Information used explicitly in decision making and can substantially change decision process. Can evolve to create (allow emerge) new behaviors and rules on the fly. | Information is used implicitly to define a static rule set. All is set once the rules are defined. Direct effects of info cannot be reversely constructed from rules. |
| Presentation | Information is used as a dimension for data analysis and presentation such as graphs. | Information measures and scales are not represented in the analysis and outcome. |

This paper seeks to emphasize the formalization of including information as a separate space in agent based models. Economic models are used as a baseline because they are common ABMs with established results over the last several decades. The focus of models in this paper is then on how to use information and how the inclusion of information affects a model. To demonstrate the benefits of AIM, it is useful to discuss levels of information applied in previous economic and agent based models and in what ways AIM will compare or diverge from them. General differences between AIM and previous model use of information can be found in Table S2. These previous models can be classified according to according to how they use information: (1) General use of information in economy theory but no ABM; (2) Used info in ABM in a way that only contribute to generate static rules; (3) Used information in an intelligent way approaching AIM.

**S4.3.1. General use of information in economic theory but not used within an ABM**:

Economic models have applied measures of information beginning in the early 1970s and many uses have been demonstrated in textbooks and literature [12,13,14,15]. A prime example is the model proposed in Akerlof's "The Market for Lemons" [16]. The market consisted of asymmetric information in which buyers could not accurately determine the value of a product before a sale is made yet the seller is able to accurately assess the value. In a two-tiered value model, there

would only be two products low-quality and high-quality (with low and high costs respectively). The buyer, unable to determine which is which would be willing to pay the average value of the product (weighted average based on expected number of low and high-quality product). Therefore, an incentive would exist for a seller to sell a low-quality product as a higher-quality product to maximze profit. The results of this model lead to the collapse of a market as higher-quality products were effectively driven out of the market because there was no benefit for the seller.

Information about quality is directly coded into the perceived or claimed value of a product. In terms of AIM, information here does not have its own dimension and is directly connected to the material space. Instead, information is a pre-determined condition expressed as a price rule in an if-then format. All interactions can be conducted with this ruleset and there is no learning and gaining information in these interactions. (Technically the buyer gains information about the value of the product after purchasing and experience or not experiencing problems with the product. However, this does not have an effect on the model.)

In AIM, there can be many more possibilities. One can define a trust variable and a trading knowledge variable in the information space to allow each party in the trade to learn and increase its own knowledge and rate how well the other can be trusted. Then Trust and knowledge can be used in future trade decisions. Agents can avoid sellers who have low trust values to prevent the overwhelming presence of fake high quality products. If the trust is removed from the system, or is not considered, then the system will behave like the simple rule based described above. But here, using AIM allows the simulation a variety of system behaviors with different levels of information usage without the need to recode the basic simulation rules (just the need to regulate the information space variables). The non-AIM method, on the otherhand, needs different sets of rules coded for each scenario.

Spence [17] examines education as a signal in the labor market. Signaling helps to counteract the effects of adverse selection (demonstrated in Akerloff's model). Signaling refers to actions taken by agents to convince other of the value or quality of their products. In these models, it is impossible to tell a high productivy work from a low productivity worker before hiring them. This is asymmetric information in the form of workers having information on how productive they are and employers not having this information. To counteract this, the employer finds the education levels of each potential hire to help guide the hiring process, with the expectation that education and productivity are correlated. Therefore, for an agent to get hired, they would like to know what education level is necessary to distinguish itself.

To do this, indifference curves can be drawn with the assumption that education is less costly for high-productivity individuals. This is done on an education-wage graph. Two curves are drawn for the education-wage combinations low and high productivity agents will find equally good as their expected education-wage pairing $(S^H, w^H)\ or\ (0, w^L)$. The low-productivity curve is steeper as these agents would wish more wages to cover the increased cost of education for them:

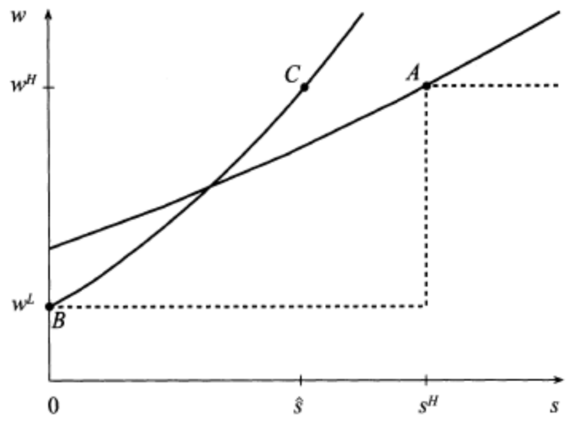


This model expands on the information concept by allowing an agent to seek to change its level of education to acquire a better paying job. However, high-productivity agents must pay for education just to signify that they are productive. High productivity agents can realize that because low-productivity agents will be less likely to pay the costs of more education, that they may require less education than initially thought (in the graph, L agents find positions B and C to be equally good, so will be more likely to choose B with no education while H agents find position C much better than A and may be likely to choose education corresponding with that).

However, this model does not allow the information to change rules or evolve with the system. Agents will get a job based on their education versus the education of other agents. This is a static rule and does not fit with an AIM model.

**S4.3.2. Used ABM and information in a minimum intelligence system**:

There exist many ABM studies which include information in some form [18,19,20,21,22,23]. However, many models which use a concept of information do not necessarily use information that would contribute to a system being classified as intelligent. Contrary to this, AIMs are generally developed to model intelligent systems.

Among the earliest ABM to include local information was Conway's Game of Life [24] which had neighboring cells share information on the state of their cell. Behavior of an individual cell was then subject to the state of neighbor cells. In terms of AIM, this concept of information does moderate the interactions between cells (life or death depending on number of alive neighbors), but the scope of information is limited to only the existence of neighboring cells and is short-lived. Information from one time-step does not affect information or physical state at another time-step. While the system does evolve physically in time, the rules regarding information of neighbors states remain constant and informaton on the previous states is lost. Essentially, this model exists only in the material space because the information used has a direct one-to-one correspondence with the material space.

Epstein and Axtell's Sugarscape model [25] expanded on Life by allowing agents to move and learn information on resources in the environment. Every step, agents gather information on the nearby cells to find nearest resources in order to survive. If an agent runs out of resources, it may die. Variations on this model allow exchange of information between nearby agents. In terms of

AIM, Sugarscape allows the learning of information about the amount of resources in the environment such that an agent will move towards the closest and largest pile of resources. This model can include interactions between agents, but the information is only a measure of the environment and not an intrinsic part of each agent. Therefore, it is an application of statewide information and all information measures don't evolve, just whether they are known or not. This is then not classified as an intelligent system because the information space itself doesn't grow.

Modifications to Sugarscape can allow the model to more fully apply available information in the system and form a more complete AIM model. For example, if each agent prioritizes close resources when nearly empty of resources, and larger stockpiles of resources when able to reach them, then each agent chooses its actions based on information about itself and the environment. This allows the agent to adjust its priorities and rules based on the combined physical and information situation. Interactions between agents which share both an agent's need of resources and information on the environment allow a complex model to emerge where agents priortize actions based on weighing their own needs versus other's needs.

Glosten and Milgrom [26] and Jacklin et. al [18]. both use information in the study of a stock market model to show effects of insider trading and portfolio insurance. In each case, information chooses which set of trading rules to apply. Informed traders know what the value of the stock, V, will be at time T and will buy stock if available below that value and sell stock if available above that value, or not trade if that value neither of the buy or sell conditions are matched. Uninformed traders do not know V and either randomly choose to buy, sell, or not trade, or they buy and sell based on the recent market trend (if stock value is increasing they buy, decreasing they sell). To actually trade, one random agent (with probability $\phi$ to be informed) per time step visits the market maker who sets buy and sell prices. The market maker, who does not know V, adjusts the stock prices between agents. The prices adjust based on the market maker's expectation of V, which is governed by the trading history observed and whether a buy or sell has just occurred. The initial expection of V is the weighted average of the possible values of V. The market maker then adjusts its ask price to the lowest price at which he will expect to break even, and the bid price to the largest price at which he will expect to break even.

The information in this model is static—an agent either knows what the end value of the stock will be or it doesn't. The market maker's information does evolve based on what has occurred, but this simply sets the prices and does not alter any other agent's rules. As such, the information used here does not fit into the realm of AIM because it is not an independent dimension (directly tied to the material value of the stock), it does not evolve, and is not an intelligent system in this context.

### S4.3.3. Used information in an intelligent way approaching AIM:

Pastore, Ponta, and Cincotti [20,21,22] study markets with similar information mechanisms to our own market model. Each model involves trading risky assets within a market. Actions of buying or selling occur each time step based on each individual's sentiments towards the market and with a probability of 0.05 (most of the time an agent will not trade). Here, sentiment is defined as the information an agent has about the market and can range from $-1 \leq S \leq 1$ which represents the portion of assets they will buy (when S is positive, the agent will buy S times the number of

stocks it can afford) or sell (when S is negative, the agent will sell S times the number of stocks it owns). This information evolves in tow ways: based on the history of the market and based on the directed random network of agents.

The history of the market examines the change in the price between this timestep and the previous timestep. The agent network is responsible for changes in an agent's sentiment. The network is directed such that interactions are uni-directional (one agent influencing another does not me the 2nd agent influences the 1st). Each interaction is characterized by a strength (each connection between agents has its own constant strength). The complete formula for evolution of information is then (h is timestep, i is each agent, $\alpha$ is weight of own sentiment, $\beta$ is weight of network sentiments, $\delta$ is weight of market behavior, $J_i$ is the i'th agent's network):

$$S_i(h+1) = F\left(\alpha_i S_i(h) + \beta_i \hat{S}_i(h) + \delta_i r(h)\right)$$

$$F(x) = \max(\min(x, +1), -1)$$

$$\hat{S}_i(h) = \frac{\sum_{k \in J_i} g_{ki} S_k(h)}{\sum_{k \in J_i} |g_{ki}|}$$

$$r(h) = \log[p(h)] - \log[p(h-1)]$$

This model does follow the methods of AIM. The authors do not explicitly examine the evolution of the information, but it would be easily accomplished based on their model.

They use a network of agents sharing information based on sentiments about the market. Our model includes this sentiments as information as well as divergent information, which may be thought of as "anti-sentiments." Our market model is attempting to demonstrate multiple information spaces in a simplistic scenario and does not include direct agent-agent interactions.

## S4.4. Model 1: Money Distribution

### S4.4.1. Methods

The example below shows an AIM model for money distribution. It does require new rules and parameters be defined to incorporate the use of information in money trading. These come in the form of redefined trading rules. The money, $M$, and information, $I$, of agent i interacting with agent j evolve according to:

$$\begin{aligned} M_i(t+\Delta t) &= M_i(t) + \Delta M_{ij} \\ M_j(t+\Delta t) &= M_j(t) - \Delta M_{ij} \\ I_i(t+\Delta t) &= I_i(t) + \Delta I_{ij} \\ I_j(t+\Delta t) &= I_j(t) + \Delta I_{ji} \end{aligned} \qquad (S69)$$

Where $\Delta M_{ij}$ and $\Delta I_{ij}$ are the change in the material and information spaces. The change in $M$ for two agents trading is equal in magnitude but opposite in direction ($\Delta M_{ij} = -\Delta M_{ji}$). The

change in information for each agent is dependent on who won the trade. $\Delta M_{ij}$ is comprised of two parts:

$$\Delta M_{ij} = P_{m_i}\left(M_i(t), M_j(t), \Delta m \, * p_{I_{ij}}\left(I_i(t), I_j(t)\right)\right) \qquad (S70)$$

Here, the function $p_{I_{ij}}$ (eq. S69) determines if the trade will occur and the winner of the trade. A normal distribution is formed around the difference between the two agents' information values. If a random value picked from this distribution is greater than the pre-defined threshold $\gamma_I$, then the trade will not occur. This protects low-information agents from participating in trades that will likely not work out well for them. Normal distributions are also formed with the information level of each agent as the means and standard deviations. A random number is chosen from these distributions and the agent with the higher number wins the trade. Note that these random numbers are generated once per attempted trade for consistency.

$$p_{I_{ij}} = \begin{Bmatrix} 0 & if\ Rand\left(\left|I_i(t) - I_j(t)\right|\right) > \gamma_I \\ 1 & else\ if\ Rand\left(I_i(t)\right) > Rand\left(I_j(t)\right) \\ -1 & otherwise \end{Bmatrix} \qquad (S71)$$

$P_{m_{ij}}$ determines how much money is traded by checking if the losing agent has enough money, $\Delta m$, for the trade to occur.

$$P_{m_{ij}} = \begin{Bmatrix} 0 & if\ M_i + \Delta m * p_{I_{ij}} < 0 \\ 0 & if\ M_j - \Delta m * p_{I_{ij}} < 0 \\ 0 & if\ p_{I_{ij}} = 0 \\ \Delta m & otherwise \end{Bmatrix} \qquad (S72)$$

With the money exchange finalized, the change in information for each agent, $\Delta I_{ij}$, can be determined based on the winner of the trade. Note that while lower case $p_{I_{ij}}$ determines the winner of trades based on information values, upper case $P_{I_{ij}}$ determines the amount of information gained by agent $i$ interacting with agent $j$.

$$\Delta I_{ij} = P_{I_{ij}}\left(p_{I_{ij}}\left(I_i(t), I_j(t)\right)\right) \qquad (S73)$$

$P_{I_{ij}}$ will always be positive and depends on if a trade is successful, who won the trade, and information values. Each agent involved in a successful trade will gain information, with the winner of the trade gaining the greater amount of information. Alternatively, an agent who cancelled a trade will gain information as well, learning from avoiding a detrimental trade while the other agent will gain less information. $\Delta I$, $\alpha_I$, and $\alpha_r$ are predetermined constants such that agents who cancel trades due to large information gaps and winners of trades gain more information than others. Other information interactions with no direct dependence on the material space are also possible, such as an agent spontaneously becoming creative and gaining a large amount of information.

$$P_{I_{ij}} = \begin{cases} \Delta I & if\ p_{I_{ij}} = 0\ and\ I_i > I_j \\ \alpha_r\, \Delta I & if\ p_{I_{ij}} = 0\ and\ I_i < I_j \\ \Delta I & if\ p_{I_{ij}} < 0\ and\ P_{m_{ij}} > 0 \\ \alpha_I\, \Delta I & if\ p_{I_{ij}} > 0\ and\ P_{m_{ij}} > 0 \\ 0 & if\ p_{m_{ij}} = 0\ and\ P_{I_{ij}} \neq 0 \end{cases} \qquad (S74)$$

To summarize, this AIM model for money distribution picks the winners of trades randomly, but with a strong preference for higher information agents. However, if the information difference is too large, low information agents may reject the trade. During a successful trade, both agents gain information with the winner gaining the larger amount. For rejected trades, the agent rejecting the trade gains a larger amount than the other agent.

### S4.4.2. Model 1: Simulation Results

The simplest information model of money distribution results in a 2D distribution with money on one axis and information on the other axis (shown in Figure S6). In this simulation, agents were given initial money and information normally distributed about ($i = 5{,}000, m = 5{,}000$) and $\Delta m$ and $\Delta i$ were kept small ($\Delta m = 5, \Delta i = 5\ or\ 10$). During each timestep, every agent attempted to initiate one trade. This way, every agent would interact during every time-step, with some agents interacting many times if randomly chosen by others. Regardless of the initial conditions, the distribution quickly transitions into a crescent moon shape (evolution into crescent shown in Figure S6 a-c). After a long time, the crescent thins out into a boomerang shape with most of the population having low information and low money (Figure S6 d).

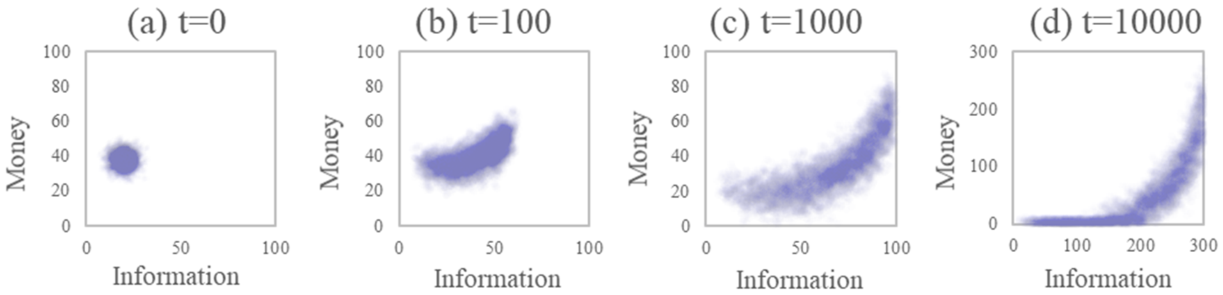


**Figure S6.** The crescent shape forms quickly from an initial normal distribution. (a) the initial distribution. (b) 100 time-steps shows the beginning of the crescent. (c) 1,000 time-steps shows the complete crescent. (d) After a long time, the crescent becomes boomerang shaped; it is thinner and most of the money is concentrated in the high information agents while low information agents have minimal money. All diagrams are scaled such that 100 money or information is the maximum value of money or information for a single agent in the crescent distribution (c). Note that the axes on the boomerang distribution extend beyond 100.

Most high money agents have high information and most low information agents have low money. This difference is not extreme while in the crescent distribution. If allowed to continue, high information agents with low money are common, while low information agents with high money are extremely rare. This pattern generally matches those seen in real economies when

comparing education level and income because higher levels of education typically result in higher incomes [27]. Information does not directly correspond to education. Education is only part of information, specifically a base level of information and the ability to gain new information. But there are no real statistical measures which correspond directly to the information definition used in this mode. Still, education may be the closest measure available.

The distribution shown in Figure **S7** does not contain extremely high information or money values. The maximum information is about $i \cong 40000$ and the maximum money is about $m \cong 15000$. Because of this, the distribution should be thought of as containing a lower-to-middle class population. However, we believe this distribution to be favorable for an entire economy such that individual agents' advantages are not as extreme as in the real world.

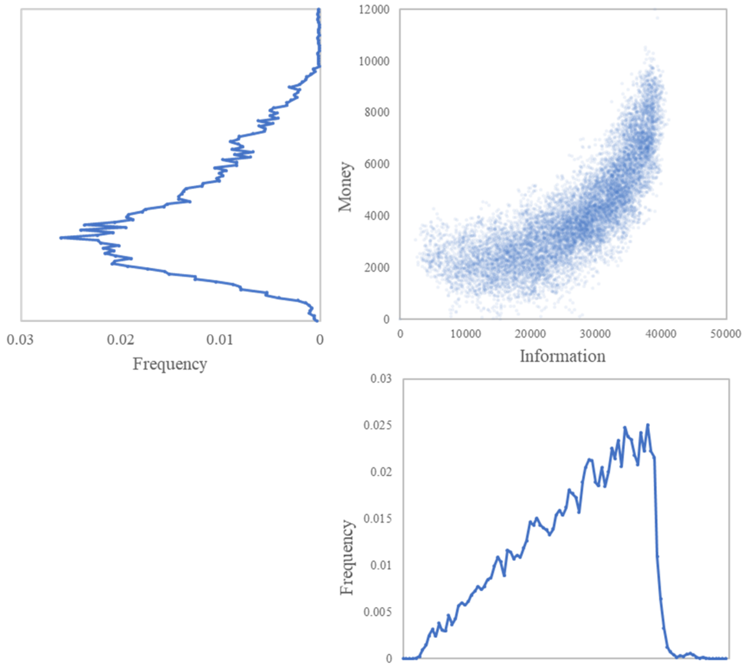


**Figure S7.** The crescent shaped information-money distribution with information and money distributions shown. Information and money are plotted as percent of the maximum single agent's information and money.

When looking at only the money distribution in this model, a shifted gamma-like distribution is seen with a long right tail and short left tail. The tail towards large money can be fit by power law distribution, with a Pareto coefficient of $\alpha = 1.5$. As the system evolves, the Pareto coefficient increases towards a maximum near $\alpha \cong 2.0$. The information distribution has a long nearly linearly increasing left tail before peaking and sharply falling off after.

The money distribution of the information model may be directly compared to previous models and real-world distributions. Basic previous models resulted in distributions with a peak at zero money and exponential decays. When adding rules to previous models such as taxation or savings, the distributions become gamma-like with a non-zero peak and the distribution is zero for zero money. The key result of these additions was the tail then fitting a power law [28].

Previous money distribution models could produce distributions comparable to real world economies. The details of these distributions emerge from initial rules and parameters

established prior to any interactions and were not representative of any single agent. By their nature, the agents in these models acted only in the material space and have a limited range of possible types of interactions without explicit and often complicated rules established.

The major difference between the distributions for the information model and previous models is the existence of a peak at non-zero money in the information model without savings or taxation considered. Adding taxation and savings to previous models develops money distributions similar to the information model.

Real world distributions typically have a small portion of the population with zero money and a major peak before the median and average incomes. These real distributions may include secondary peaks showing a separation between a large rich and poor populations. One of the key real-world distribution characteristics is the existence of a power law tail for high money values [29].

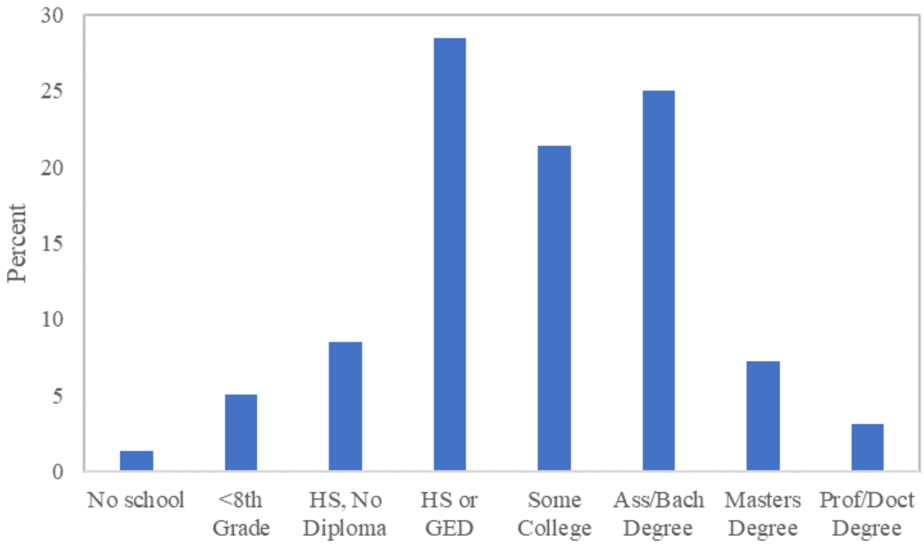


**Figure S8.** US education distribution for 25-year-old and over residents.

The information model distribution resembles real world distributions. Specifically, this model is strikingly similar to the UK disposable income distribution from 2016 (Figure S9). Both contain a region of low money containing a very small portion of the population. Next, a sharp peak is observed before the mean money value. Third, both contain long Pareto law tails.

Both the information model and previous models partially produce the real-world distribution. Without the introduction of additional rules such as taxation and savings, the information model is significantly closer to forming the real-world distribution than previous models. With the additional rules, the previous models have similar characteristics to the information model.

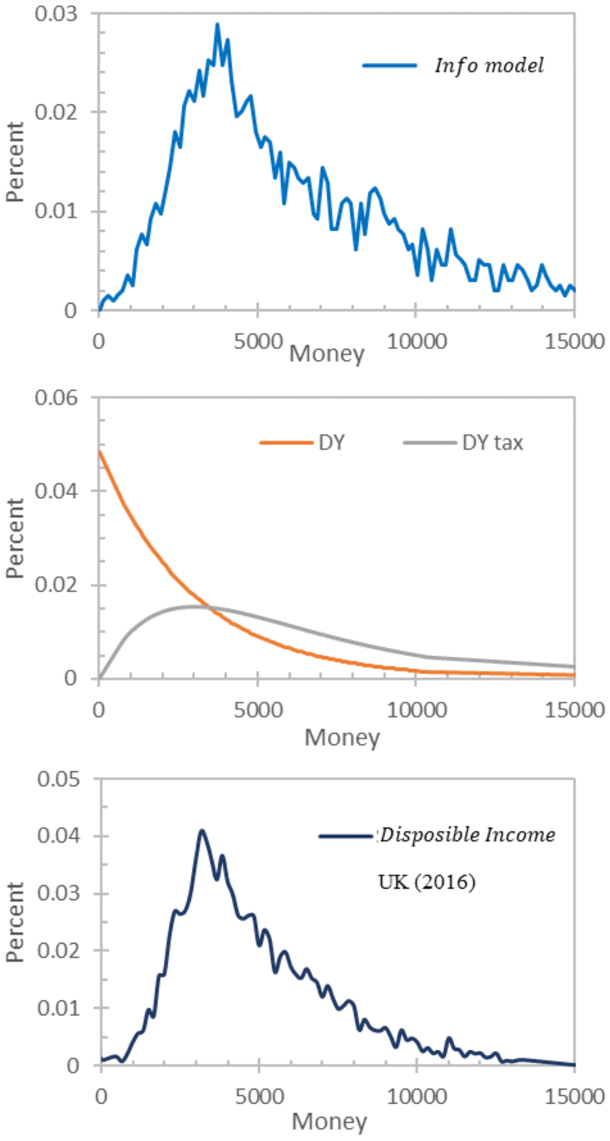


**Figure S9.** Money distribution. (a) the information model, (b) the Dragulescu & Yakovenko model[15], and (c) the disposable income distribution from the UK 2016 [30]. The percent of the population with money m is plotted. The DY model and UK data are scaled to have an average amount of money equal to the information model (5,000).

## S4.5. Model 2: Sudden stimuli applied to money distribution

### S4.5.1. Model 2a: Simulation results

The average and extreme trajectories of these agents are plotted in information-money space in Figure S10 (grant in blue, lottery in green, and creativity in red), along with the crescent shaped distribution (very light blue in background). The pre-stimulus expected trajectories are plotted in lighter colors (pre-grant in light blue, pre-lottery in light green, and pre-creativity in light red). These are connected to the post-stimulus agents by a dashed line. The average path of

agents under each effect is plotted using a bold line. Extreme trajectories are potted with dashed lines. These include the maximum and minimum money values for each set of agents.

Examining Figure S10, both the green (creativity) and blue (grant) trajectories look qualitatively similar. Their maximum, minimum, and mean trajectories indicate similar changes to information and wealth for these agents. The primary difference is that red starts at about a quarter of the money that blue does. This implies that initial money does not have a significant role in determining the success of an agent. Based on the defined information interaction rules, this is expected. This happens because it is the information space that moderates who wins trades. Agents with similarly high information are similarly likely to win trades with other agents.

Green (lottery) does not perform as well. The green average indicates that agents move toward the crescent distributions. The minimum demonstrates a sharp decrease in money with little change in information. This is contrary to the high information blue and red minimum trajectory agents which had much larger information changes. The maximum green trajectory increases money by far less than blue and red maximum trajectories. Altogether this points toward an important condition to satisfy to encourage success — high information is required to maximize odds of success. This is the qualitative answer the venture capitalist was interested in.

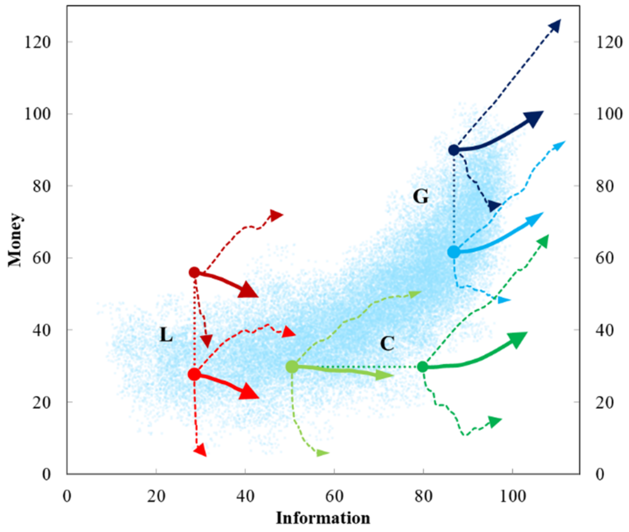


**Figure S10.** Trajectories of agents under grant (blue), lottery (green), and creativity (red) conditions. The crescent distribution is shown in yellow. Each modified agent attempts 1,000 trades. The simulation is repeated 100 times (boosting the same agents each time). Starting points for the trajectories are the money and information values of the agent after the grant, lottery, or creativity has been applied. These points are signified by the circles of appropriate color. This chart displays the average (thickest lines), minimal, and maximal trajectories. The scale in this diagram is percent of the original maximum information (horizontal) and money (vertical) of an individual agent in the crescent.

The average, minimum, and maximum changes to money and information are found in Table S3. Grant and creativity agents had nearly identical maximum and minimum money changes and average money changes that were not significantly different from each other ($t(198) = 1.25$, $p = 0.21$). Both grant and creativity money change averages were significantly higher than those for

lottery agents (respectively: $t(198) = 18.9$, $p < 0.001$; $t(198) = 12.3$, $p < 0.001$). The average information change for each group of agents was significantly different from each other (grant vs. lottery: $t(198) = 9.95$, $p < 0.001$; grant vs. creativity: $t(198) = 8.01$, $p < 0.001$; lottery vs. creativity: $t(198) = 15.8$, $p < 0.001$). The creativity agents' minimum, average, and maximum information change is ~3.4 higher than the grant agents'. This is likely due to two causes: (1) the creativity agents' initial information is 7.0 lower than grant agents', and (2) agents with information levels larger than the crescent's maximum information will have few agents willing to trade with them and therefore have slower information gains.

**Table S3.** Changes to money and information.

| | Money Change | | | Info Change | | |
|---|---|---|---|---|---|---|
| | Min | Avg (SE) | Max | Min | Avg (SE) | Max |
| Grant | -16.0 | 11.0 (0.3) | 36.4 | 11.6 | 20.3 (0.3) | 26.4 |
| Lottery | -23.1 | -6.9 (0.9) | 17.2 | 3.0 | 14.5 (0.5) | 25.5 |
| Creativity | -15.9 | 9.7 (1.0) | 36.8 | 15.1 | 23.7 (0.3) | 29.7 |

The general behavior of agents under each condition does not tell how likely it is for agents to succeed, just that either they are or are not likely to. Therefore, it is beneficial to examine how likely it is for the agents to have significant changes to money and information. These are summarized in Table S4 below. All percentages are out of the 100 agents subjected to each stimulus. Significant changes are considered 10% in money and 20% in information to keep the range for minimal change at 20% for each (-10% to 10% in money, 0% to 20% in information).

The trajectories of grant winners show a variety of behaviors of their money (blue in Figure S10). On average, their money and information increase gradually over the course of 1000 attempted trades. Some agents increase money and information quickly, while few lose money.

High information agents are likely to succeed due to the defined rules governing interactions between money and information. Data on the changes to information and wealth of agents can be found in Table S3 and Table S4. It comes as no surprise that 60% (58% increase information + 2% minimal information change) of agents awarded a grant (blue in Figure S10), who already have high information, increased their money further. Additionally, 38% of agents (25% increased information + 13% minimal information change) maintain their level of money. Just 2% of agents lost more than 10% of their money and each of these agents had minimal information change. Agents who won a grant and gained little information were likely to gain little money as well (76%, 13/17). This subset of agents who gained little information were just as likely to lose money as to gain money (12%, 2/17).

Behavior of grant winners in this model may be compared to similar real life scenarios. For example, if similar research groups are awarded identical grants for their experiments, then it is likely that will increase their information and money. This is not guaranteed as demonstrated in this model. Despite their advantages over much of the distribution's population, many agents (38%) maintain their level of money. A few even lose money (2%).

This model is a simplified simulation in which complex behavior emerges from the interaction between information and material dimensions. Completely modeling real life system may require additional dimensions and/or specific rules.

**Table S4.** Percent of agents with money and information change.

| | Info Change | Money Change | | |
|---|---|---|---|---|
| | | Decrease | Minimal | Increase |
| Grant | Increase | 0 | 25 | 58 |
| | Minimal | 2 | 13 | 2 |
| Lottery | Increase | 55 | 33 | 8 |
| | Minimal | 3 | 1 | 0 |
| Creativity | Increase | 12 | 13 | 74 |
| | Minimal | 0 | 1 | 0 |

Lottery winners (green in Figure S10) are agents who are lucky and gained a significant amount of money. To differentiate this group from the grant winners, these agents are restricted to agents originating in the lower half of the information space. After gaining the lottery money, these agents typically lost significant portions of their money, falling back into the regular crescent distribution.

Most of these agents (55%) lost wealth while their information increased and 3% lost wealth with minimal information change (Table S4). A sizable portion of lottery winning agents (34%, 33% with information increase + 1% minimal information change) managed to keep their new money. Only 8% of lottery winning agents could increase their money (each of these agents also increased their information). The magnitude of the money increase was less than half of the maximal creative and grant agents.

The pattern of significant money loss by lottery winners fits well with anecdotal stories of real lottery winners losing most of their new-found money in a relatively short period. Without learning how to deal with the large economic change (e.g. increasing information through training), these people and agents have a decreased chance to maintain their new economic status.

Creative agents tend to remain some of the highest-information agents and are likely to increase their money. Qualitatively these agents act similar to grant agents. The magnitude of their money changes is similar. Creative agents tend to have slightly larger information gains. Most of these agents (74%) increased both their money and creativity (Table S4). The remainder were split evenly between maintaining (13%) and losing (12%) money. Only 1 agent had minimal information change (this agent also had minimal money change).

Tying this simulation to a real-life situation, the information boosted agents (red in Figure S10) resemble the first few miners learning of a new location with large quantities of gold in the ground. Miners with identical initial money and information levels will have varying levels of success. Most will succeed, but some are not as lucky.

Overall, the creativity and grant agents accrue similar levels of money and information increases despite the differences in their initial money. Lottery agents, however, tend to lose their newfound money. Returning to the example of a venture capitalist, the smart investment would be in an agent with high information to maximize chances of success. However, creative agents are more likely to gain money than grant agents (74% and 60% respectively). In addition, creative agents are more likely to lose money than grant agents (12% and 2% respectively). This is likely caused by the difference in starting money. Grant agents require larger changes in money than creative agents to see significant increases or decreases.

Previous models had no method to study the sudden increase in an agent's information. Instead, they would need to add a new rule making a specific agent perform better than others. In contrast, the information model allows this behavior to emerge spontaneously. An agent is given stimuli and the predefined rules allow its evolution. These rules were defined in the first model and were not altered for this model.

### S4.5.2. Model 2b: Simulation Results

Additionally, this model allows the long-term trajectory of agents to be recorded. For example, a single agent was simulated 2000 times with the same initial parameters as the pre-lottery winning agents. The behavior over 5000 time-steps is split into three representative groups and is shown in Figure S11. In the figure, the average behavior of the groups is shown using three red lines. In addition, the distribution of agents in the long-term distribution are plotted in green. These groups represent levels of economic class changes.

The best performing group gains money and information quickly. These agents can be thought of as improving their class. It is likely that these agents continue to improve as time increases. The second group loses money in exchange for information, but ends up with a similar amount of money as they started with. These agents maintain their class. The third group of agents quickly loses money and must gain information to stabilize their money level.

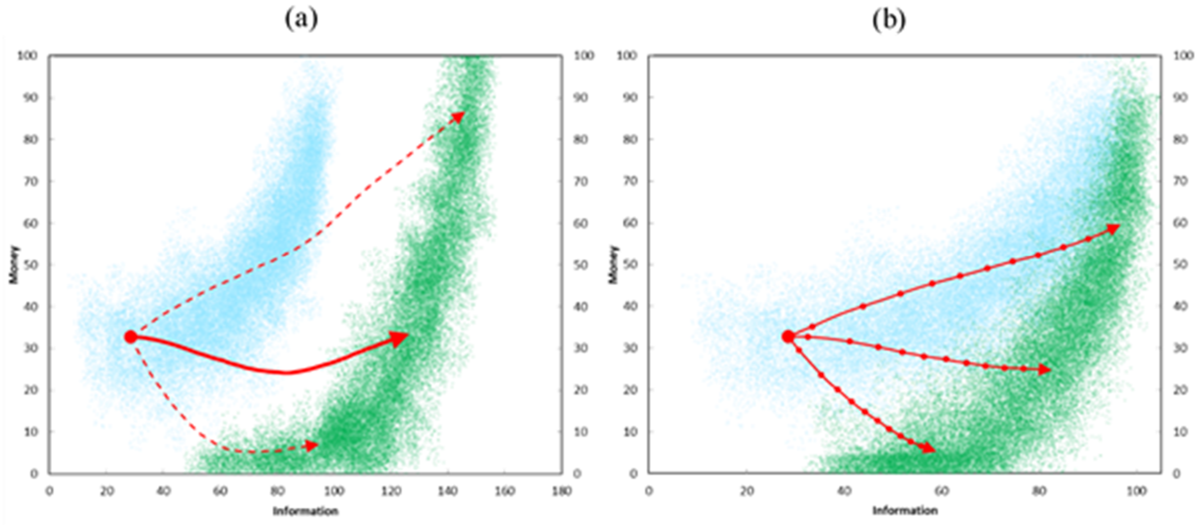


**Figure S11.** Possible long-term behavior of a single agent. (a) Maximum, average, and minimum agent trajectories. (b) The three red lines represent average trajectories of the agent when falling into three levels of class change. The green distribution is the final distribution of the single agent simulation repeated 2000 times. The simulations in (a) ran twice as many time-steps as (b), which explains the difference in separation between the green distributions and blue distributions.

In this diagram (Figure S11) levels of information change are key to explaining the different behaviors. Low, moderate, and high information gains are directly related to money loss, little money change, and money gain respectively. Agents gaining money generally follow the crescent shape, while other agents fall out of the crescent either quickly (money loss agents), or slowly (little money change agents).

## S4.6. Model 3: Stock model

### S4.6.1. Methods

Both the material space and information space will consist of two variables: money, $m$, and stocks, $s$, in material space, $M$, and normal information, $i$, and divergent information, $d$, in information space, $I$. As such, each space can be represented by vectors:

$$M_i(t) = \begin{pmatrix} m_i(t) \\ s_i(t) \end{pmatrix}$$
$$I_i(t) = \begin{pmatrix} i_i(t) \\ d_i(t) \end{pmatrix} \quad (S75)$$

Where the i'th agent has money $m_i$, stocks $s_i$, normal information $i_i$, and divergent information $d_i$. Each space evolves after considering their material and information spaces, history of stock prices $H(t)$, news $N(t)$, and stocks available $S(t)$. The information space will have intermediate values to be used during this time-step. These are notated using primes (i.e. $I_i \rightarrow I_i', i_i \rightarrow i_i', d_i \rightarrow d_i'$)

$$I'_i(t) = I_i(t) + f_{I_i}(H_i(t)) + N(t) \quad (S76)$$

$$M_i(t + \Delta t) = M_i(t) + \Delta M_i(M_i(t), I_i'(t + \Delta t), S(t)) \quad (S77)$$

The history of stock prices $H(t)$ contains all historical stock prices. Each agent individually chooses how to apply this to their information space. The news $N(t)$ applies equally to all agents. In addition to the trading agents in this model, a single market agent and a single news agent are added which interact with the system differently than trading agents.

First, agents adjust their information space. The amount of information change depends on an individual agent's views on stock history (i.e. how long in the past to look at stock prices is time $T$,and how strongly an agent considers deviation from mean price when deciding whether to buy or sell):

$$\mathrm{f}_{\mathrm{I_i}}(t) = \alpha_{i_1}(p(t) - p(t - T)) + \alpha_{i_2}(p(t) - \overline{p(t - T{:}\, t)}) \quad (S78)$$

Where $\alpha_i$'s are constant vectors specific to each agent, $p(t)$ is the price at time $t$, and $\overline{p(t - T{:}\, t)}$ is the average price over the time interval $t - T$ to $t$. If $f_{I_i}(t) < 0$ in either its normal information component or divergent information component, then that component is set to zero such that information space never decreases. The news affects the information space of all agents equally and bases its value on the state of the system and history of stock prices. It acts similar to individual agents, but applies its effect to all agents:

$$N(t) = \alpha_{N_1}(p(t) - p(t - T_N)) + \alpha_{N_2}(p(t) - \overline{p(t - T_N{:}\, t)}) \quad (S79)$$

This completes the information change. Before change in the material space can occur, agents must now choose whether to buy, sell, or hold stocks. This is accomplished by finding the difference between two random numbers centered on an agent's normal information and divergent information.

$$b_i(t) = \frac{\left(Rand(i_i'(t)) - Rand(d_i'(t))\right)}{|i_i'(t) - d_i'(t)| + 1} \quad (S80)$$

If this value is over a threshold $|b_i| > \alpha_b$, the agent will attempt to buy ($b_i > 0$) or sell ($b_i < 0$). Values larger than 1 or less than -1 are set to 1 and -1 respectively. If not over the threshold, the agent will hold their stocks. Each buying agent selects a maximum price to buy stocks at and a maximum number of stocks to buy. Constraints are placed so that an agent cannot spend more money than owned. Each selling agent selects a price to sell stocks at and how many stocks to sell. A constraint is placed so that agents will not try to sell more stocks than they own.

Agents choose prices based on $b_i$ and the current price $p(t)$, with the constraint that this price must be within the range $p(t) * (1 \pm \alpha_p)$, with $\alpha_p$ a constant for all agents.

$$p_i(t) = p(t) * \left(1 + \alpha_p * Rand\big(b_i(t)\big)\right) \qquad (S81)$$

After determining the price, agents must choose how many stocks to buy or sell. Here, $b_i$ acts as the fraction stocks owned or able to buy that an agent will attempt to sell or buy. $n_i(t)$ is the number of stocks an agent will attempt to buy ($n_i > 0$) at or below price $p_i$, or sell ($n_i < 0$) at price $p_i$.

$$n_i(t) = \left\{ \begin{matrix} b_i(t)\dfrac{m_i(t)}{p_i(t)} & if\ b_i > 0 \\ b_i(t)s_i(t) & if\ b_i < 0 \end{matrix} \right\} \qquad (S82)$$

The market tracks and orders agents selling stocks in $S(t)$. Then, agents buying stocks will be chosen at random to attempt to buy. If sellers have stocks available for price(s), $p_j$, under the buyer's maximum price $p_i$, the buyer will buy as many as possible up to its maximum number to buy $n_i$. Partial orders are filled to the extent possible. The number of stocks gained by an agent is $\Delta s_i$ and the amount of money gained by an agent is $\Delta m_i$.

$$\Delta M_i = \begin{pmatrix} \Delta m_i \\ \Delta s_i \end{pmatrix} \qquad (S83)$$

After all agents looking to buy have attempted to buy stocks, the market closes and unsold stocks are returned to their owners. The price of the stock is adjusted to the highest priced stock sold during this time-step. If no stocks are sold, the stock price decreases to:

$$p(t + \Delta t) = p(t) * \left(1 - \alpha_p\right) \qquad (S84)$$

After each time-step, normal information and divergent information are normalized for each agent.

$$\begin{aligned} i_i(t + \Delta t) &= \frac{i_i'(t)}{i_i'(t) + d_i'(t)} * 100 \\ d_i(t + \Delta t) &= \frac{d_i'(t)}{i_i'^{(t)} + d_i'(t)} * 100 \end{aligned} \qquad (S85)$$

Without normalization, eventually the changes to the information space would become insignificant compared to the information totals. Effectively, this forces all absolute information changes to become percent changes and consider recent information changes.

The simulation results are detailed in the main text.

**Summary of Variables Defined and Used**

**Table S5.** Constants and variables related to physical discussions in this paper.

| Variable | Definition |
|---|---|
| $\alpha$ | Distribution constant |
| $a$ | Side-length of box |
| $\beta$ | $1/k_bT$ |
| $d$ | Diameter of particles |
| $E$ | Energy of system |
| $f(v)$ | Velocity distribution |
| $\bar{f}$ | Frequency of collisions for a single particle |
| $F$ | Frequency of collisions for a system |
| $F_{pp}$ | • Between particles |
| $F_w$ | • With walls |
| $\hbar$ | Planck constant divided by $2\pi$ |
| $h_x$ | Differential entropy |
| $I$ | Impulse |
| $k$ | Wave number |
| $k_b$ | Boltzmann constant |
| $l$ | Mean free path |
| $m$ | Mass of particle |
| $n_c$ | Number of collisions |
| $N$ | Number of particles |
| $p$ | Momentum |
| $p_0$ | Initial momentum of incoming particle |
| $p_T$ | Sum of particle momenta |
| $P$ | Pressure |
| $Q$ | Heat |
| $R$ | Radius of particles |
| $S$ | Entropy |
| $\tau$ | Average time between collisions |
| $t$ | Time |
| $T$ | Temperature |
| $U$ | Internal Energy |
| $V$ | Volume |
| $v$ | Velocity of particles, or speed when denoted |
| $v_0$ | Initial velocity of incoming particle |
| $v_T$ | Sum of particle speeds |
| $W$ | Work |
| $\gamma$ | Euler-Mascheroni Constant |
| $\lambda_{th}$ | Thermal wavelength |

**Table S6.** List and description of constants and variables related to local information.

| Variable | Definition |
|---|---|
| $\alpha$ | Various coefficients |
| $b_i$ | Individual demand |
| $d$ | Divergent information |
| $\varepsilon$ | Information energy |
| $H$ | Shannon information |
| $H(t)$ | History of stock prices at time $t$ |
| $h$ | Per unit entropy |
| $h_x$ | Differential Entropy |
| $I$ | Information space |
| $i$ | Normal information (Local normal information) |
| $L$ | Local information |
| $m$ | Money |
| $M$ | Money space |
| $N$ | Total number of agents |
| $N(t)$ | $News$ at time $t$ |
| $n$ | |
| $P_I$ | Final information change |
| $P_M$ | Final money change |
| $p(t)$ | Current price (of stocks) |
| $p_I$ | Function determining winner of trade |
| $S$ | Entropy |
| $S(t)$ | Stocks available at time $t$ |
| $s$ | Stocks |
| $\sigma$ | Reduced entropy |

**References**


1. Lambert, F. L., Configurational Entropy Revisited. *Journal of Chemical Education* **84** (9), 1548-1550 (2007).

2. Lambert, F. L., The conceptual meaning of thermodynamic entropy in the 21st century. *International Research Journal of Pure & Applied Chemistry* **1** (3), 65-68 (2011).

3. Martyushev, L. M., Entropy and Entropy Production: Old Misconceptions and New Breakthroughs. *Entropy* **15**, 1152-1170 (2013).

4. Martin, J. S., Smith, N. A. & Francis, C. D., Removing the entropy from the definition of entropy: clarifying the relationship between evolution, entropy, and the second law of thermodynamics. *Evolution: Education and Outreach* (2013).

5. Lambert, F. L., Disorder-A cracked crutch for supporting entropy discussions. *Journal of Chemical Education* **79** (2), 187-192 (2002).

6. Huang, K., *Statistical Mechanics (2nd edition)* (Taylor and Francis, New York, 1987).

7. Shannon, C. E. & Weaver, W., *The mathematical theory of communication* (University of Illinois Press, Urbana, 1949).

8. Jaynes, E. T., Information theory and statistical mechanics. *Physical Review* **108**, 171-190 (1957).

9. Jaynes, E. T., Prior Probabilities. *IEEE Transactions On Systems Science and Cybernetics* **4** (3), 227-241 (1968).

10. Jaynes, E. T., *Probability Theory: the Logic of Science* (Cambridge University Press, Cambridge, 2003).

11. Michalowicz, J. V., Nichols, J. M. & Bucholtz, F., *Handbook of Differential Entropy* (CRC Press, Boca Raton, 2014).

12. Birchler, U. & Bütler, M., *Information Economics* (Routledge, New York, 2007).

13. Brunnermeier, M. K., *Asset pricing under asymmetric information: bubbles, crashes, technical analysis, and herding* (2001).

14. Löfgren, K.-G., Persson, T. & Weibull, J. W., Markets with Asymmetric Information: The Contributions of George Akerlof, Michael Spence, and Joseph Stiglitz. *The Scandinavian Journal of Economics* **104** (2), 195-211 (2002).

15. O'Hara, M., *Market Microstructure Theory* (Blackwell Publishers, Cambridge, MA, 1995).

16. Akerlof, G. A., The Market for 'Lemons': Quality Uncertainty and the Market Mechanism. *Quarterly Journal of Economics* **84** (3), 488-500 (1970).

17. Spence, M., Job Market Signalling. *The Quarterly Journal of Economics* **87** (3), 355-374 (1973).

18. Jacklin, C. J., Kleidon, A. W. & Pfeiderer, P., Underestimation of Portfolio Insurance and the Crash of October 1987. *The Review of Financial Studies* **5** (1), 35-63 (1992).

19. Chakraborti, A., Toke, I. M., Patriarca, M. & Abergel, F., Econophysics Review: II. Agent-based models. *Quantitative Finance* **11** (7), 1013-1041 (2011).

20. Pastore, S., Ponta, L. & Cincotti, S., Heterogeneous information-based artificial stock market. *New J. Phys.* **12**, 053035 (2010).

21. Ponta, L., Pastore, S. & Cincotti, S., Information-based multi-assets artificial stock market with heterogeneous agents. *Nonlinear Analysis: Real World Applications* **12**, 1235 (2011).

22. Ponta, L., Pastore, S. & Cincotti, S., Static and dynamic factors in an information-based multi-asset artificial stock market. *Physica A* **492**, 814-823 (2018).

23. Macal, C. M. & North, M. J., Tutorial on agent-based modelling and simulation. *Journal of Simulation* **4**, 151-162 (2010).

24. Gardner, M., The fantastic combinations of John Conway's new solitaire game "Life". *Scientific American* **223**, 120-123 (1970).

25. Epstein, J. M. & Axtell, R., *Growing Artificial Societies: Social Science from the Bottum Up* (MIT Press, Cambridge, MA, 1996).

26. Glosten, L. & Milgrom, P., Bid, ask, and transaction prices in a specialist market with heterogeneously informed traders. *Journal of Financial Economics* **14**, 71-100 (1985).

27. Bingley, P. & Martinello, A., Measurement error in the Survey of Health, Ageing and Retirement in Europe: A validation study with administrative data for education level, income and employment. *SHARE Working Paper Series* **16-2014**, 1-33 (2014).

28. Chatterjee, A. & Chakrabarti, B. K., Kinetic exchange models for income and wealth distributions. *European Physical Journal B* **60**, 135-149 (2007).

29. Richmond, P., Hutzler, S., Coelho, R. & Repetowicz, P., in *Econophysics and Sociophysics* (Wiley-VCH, Weinheim, Germany, 2006), pp. 131-159.

30. Office for National Statistics, 2017.
